# Climate‐Driven Range Shifts and Conservation Challenges for Brown Bears in Türkiye

**DOI:** 10.1002/ece3.71019

**Published:** 2025-04-01

**Authors:** Ercan Sıkdokur, İsmail K. Sağlam, Çağan H. Şekercioğlu, Irfan Kandemir, Ali Onur Sayar, Morteza Naderi

**Affiliations:** ^1^ Department of Molecular Biology and Genetics Koç University Istanbul Türkiye; ^2^ School of Biological Sciences University of Utah Salt Lake City Utah USA; ^3^ KuzeyDoğa Derneği, Istasyon Mahallesi Ismail Aytemiz Caddesi Kars Türkiye; ^4^ Department of Biology Ankara University Ankara Türkiye; ^5^ Department of Forestry Çankırı Karatekin University Çankırı Türkiye; ^6^ Department of Biology, Faculty of Sciences Sakarya University Sakarya Türkiye

**Keywords:** Brown bear ( *Ursus arctos* ), climate change, habitat suitability, large carnivores, mammal conservation, protected areas, Türkiye, wildlife ecology

## Abstract

Understanding the current and future distribution of wildlife species is crucial for effective conservation planning, particularly in the face of climate change and increasing anthropogenic pressures. This study aims to assess the potential distribution of brown bears across Türkiye both presently, by 2050 and 2070, considering various climate change scenarios, evaluating habitat vulnerability, and reassessing the effectiveness of protected areas. Using an ensemble forecasting approach, we modeled brown bears' current and future habitat suitability, incorporating 608 occurrence records along with bioclimatic, topographic, and anthropogenic predictors under climate scenarios. Our model estimates that approximately 17.3% of Türkiye (135,556 km^2^) currently offers suitable habitat for brown bears with the highest suitability found in the Euro‐Siberian (46%), Irano‐Turanian (43%), and Mediterranean (11%) biogeographic regions. The results indicate that climate change combined with anthropogenic pressures, is expected to reduce brown bear habitat suitability by 40%–48% by 2050, and 40%–67% by 2070 under various scenarios. A significant contraction in the brown bears' range, along with a northward shift in suitable habitats, is projected, reflecting the broader impacts of climate. Additionally, the suitability of brown bear habitats is estimated to be strongly influenced by the changes in altitude. The proportion of suitable habitats under protection is projected to decline from 21.4% to 15%–16.1% by 2050 and further to 11.3%–15.9% by 2070, depending on the scenario. These findings highlight the need for targeted conservation strategies to address the emerging conservation gap for brown bears in the Mediterranean, Irano‐Turanian, and Euro‐Siberian regions. Enhancing connectivity between fragmented habitats and reassessing the status of protected areas are critical actions to safeguard the brown bear population in Türkiye. This study underscores the pressing conservation challenges and strategic opportunities for securing the future of brown bears in Türkiye.

## Graphical abstract

This study investigates the potential distribution of brown bears ( *Ursus arctos* ) in Türkiye under various climate change scenarios, highlighting significant habitat vulnerability and a projected northward range shift. By employing an ensemble forecasting approach, we assess the efficacy of existing protected areas and emphasize the urgent need for enhanced conservation strategies to mitigate the impacts of climate change and habitat fragmentation on this keystone species.

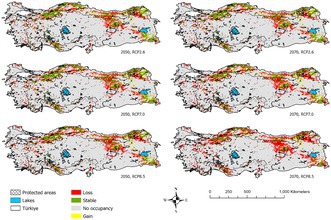

## 1 Introduction

Rapid industrialization, urban expansion, continuous population growth have led to two pressing global challenges: climate change and the biodiversity crisis (Urban 2015). Projections suggest that many plant and animal species will undergo dramatic range shifts and face extinction if global temperatures continue to rise at the current rate compared to pre‐industrial times (IPCC, 2023). Concurrently, widespread human land use is altering land‐cover extensively, leading to notable habitat degradation (Powers and Jetz 2019). Activities such as deforestation are key drivers behind the loss of habitats for countless species (Tucker et al. 2018). Large carnivores are particularly vulnerable to habitat reduction and higher extinction risk due to their reliance on expansive, intact landscapes (Wolf and Ripple 2017). Climate change and habitat loss disrupt many critical aspects of a species' viability, such as feeding, denning, and reproductive areas (González‐Bernardo et al. 2020; Penteriani et al. 2019). The changes in species' behavior, distribution, and abundance of large carnivores have profound side effects on the entire ecosystem (Dar et al. 2021; Martínez Cano et al. 2016; Zarzo‐Arias et al. 2019). Shifts in bioclimatic conditions and changes in land‐use patterns also can result in reduced populations of natural prey for predators (Pérez‐Girón et al. 2024; Petherick et al. 2021), forcing species to disperse into new areas as vegetation and habitat composition change (Penteriani et al. 2019; Pérez‐Girón et al. 2024). These interactions often lead to heightened human–wildlife conflict (Bombieri et al. 2019; Sıkdokur et al. 2024), which can hinder species' ability to adapt and pose significant challenges to conservation initiatives (Khosravi et al. 2021; König et al. 2021; Farahani and Asgharzadeh 2023). As a result, understanding both the present and potential future distribution of species and habitat vulnerability is key to developing successful conservation strategies. Ensuring connectivity between existing and potential suitable habitats is vital for facilitating gene flow and preserving genetic diversity among species (Ashrafzadeh et al. 2018; Mohammadi et al. 2021). Species distributions are influenced by both biotic and abiotic factors and shaped by their mobility within geographical and environmental spaces (Soberon and Peterson 2005). Ecological Niche Modeling (ENM) is a powerful tool that helps explain how these elements impact species distributions. By defining environmental space through related variables, ENM projects these patterns onto geographic areas (Sillero et al. 2021). This approach provides essential insights into habitat vulnerability, connectivity, and the anticipated effects of climate change.

Türkiye, a mountainous country in Southwest Asia, is a crucial land bridge linking the eastern Palearctic region to Europe. Its diverse topography, plant communities, and climatic zones offer rich habitats for numerous animal species. Türkiye is home to three of the world's 36 biodiversity hotspots: the Caucasus, Irano‐Anatolian, and Mediterranean basin (Şekercioĝlu et al. 2011; Gür 2022). However, climate change is expected to have severe impacts on the country in the near future (Önder et al. 2009; Ozturk et al. 2015; Demircan et al. 2017; Newbold et al. 2020). Türkiye's landscape is divided into three distinct biogeographic regions—Euro‐Siberian, Irano‐Turanian, and Mediterranean—each characterized by unique climatic and topographic features. Though transition zones exist between them, these regions vary in vegetation, geography, and land use (Noroozi et al. 2019). Climate change is anticipated to affect these regions differently (Ozturk et al. 2015; Demircan et al. 2017). Eurasian brown bear (Ursus arctos arctos), the only bear species that live in Türkiye, inhabit all three biogeographic regions, albeit with varying density (Ambarlı et al. 2016). Over the past two decades, Türkiye has faced an escalating biodiversity crisis (Şekercioglu et al. 2011). Activities such as mining, tourism, construction of power plants and dams, and increasing road infrastructure have greatly contributed to habitat degradation (Bilici and Akay 2021; Şekercioglu et al. 2011; Atmiş et al. 2024). Despite its biological richness, Türkiye grapples with conservation issues, as only 13.7% of the country's land is protected, with a mere 7.8% being terrestrial (NCNP 2022). Consequently, protected areas remain below international conservation targets (CBD; http://www.cbd.int/sp/targets), making the efforts largely symbolic due to the inadequate enforcement of protection measures.

Brown bear ( *Ursus arctos* ), Türkiye's largest carnivore, inhabits diverse ecological niches across the country. Their habitats range across diverse elevations, including mixed shrublands, coniferous forests, broad‐leaved woodlands, and open rocky/barren terrains. Due to their ecological importance and key trophic role, brown bears are ideal for scaling up localized habitat models to address broader conservation challenges (Scharf and Fernández 2018). However, as slow‐reproducing animals with large body sizes, brown bears are projected to be highly vulnerable to climate change (Ashrafzadeh et al. 2022; Dai and Peng 2021; Dar et al. 2021; Penteriani et al. 2019; Su et al. 2018). In addition, the human‐brown bear conflict across the country has increased in recent years as a result of the decrease in bears' habitat size and quality (Sıkdokur et al. 2024). As their preferred habitats diminish, brown bears, which show relatively high tolerance to human presence, often venture near human settlements, agricultural lands, and pastures (Cozzi et al. 2016; Kemahlı et al. 2023; Zarzo‐Arias et al. 2021). Despite this, there remain limited ecological studies on brown bears in Türkiye (Ambarlı et al. 2016, 2018; Ambarlı 2016; Can and Togan 2004; Çilingir et al. 2016; Cozzi et al. 2016; Kemahlı et al. 2023; Suel 2019; Tavşanoğlu et al. 2021), and no comprehensive research has been conducted on their habitat suitability across the country. For all these reasons, understanding the current distribution of brown bears and assessing how climate change will affect their habitat suitability is critical for future conservation approaches. This study sets out to achieve three main goals: (i) identify the current potential distribution of brown bears in Türkiye; (ii) project and assess their potential habitat suitability under different CO_2_ emission scenarios for the 2050s and 2070s; (iii) reevaluate the effectiveness of existing protected areas in conserving brown bear habitats under both present and future conditions.

## 2 Material and Methods

### 2.1 Study Area

With an area of 783,562 km^2^ Türkiye, located in southwestern Asia, serves as a key land bridge between the Eastern Palearctic and Europe. The country is characterized by diverse mountain ranges, including the North Anatolian Mountains and the Taurus Mountains to the south, which form significant barriers that separate the inner and coastal regions. The topography is highly rugged, with altitudes ranging from below sea level (−2 m) to the highest peak, Mount Ararat (5137 m), with an average elevation of 1130 m. It encompasses three of the world's 36 biodiversity hotspots: the Caucasus, Irano‐Anatolian, and Mediterranean (Şekercioglu et al. 2011; Gür 2022). These hotspots are characterized by distinct vegetation, climate, and ecological conditions, which provide critical habitats for brown bears and other species. Türkiye's climate is varied, with temperate, continental, and Mediterranean zones, each influencing the habitats available to wildlife. Climate change is expected to impact the regions differently, with temperature increases predicted in the coming decades (Ozturk et al. 2015; Demircan et al. 2017). Türkiye is divided into three major biogeographic regions: the Euro‐Siberian, Irano‐Turanian, and Mediterranean basin (Noroozi et al. 2019; Gür 2022).

The Mediterranean climate dominates the Mediterranean biogeographic region, marked by dry summers, mild and rainy winters, and irregular rainfall patterns. From sea level to higher elevations, the landscape transitions from maquis and Turkish pine forests ( *Pinus brutia* ) to black pine ( *Pinus nigra* ) and cedar forests ( *Cedrus libani* ). Additional tree species in this region include pine ( *Pinus pinea* ), Taurus fir (*Abies cilicica*), Mediterranean cypress ( *Cupressus sempervirens* ), junipers (*Juniperus* sp.), common yew ( *Taxus baccata* ), hawthorn (*Crataegus* sp.), and oak (*Quercus* sp.) species. At higher altitudes, the tree line gives way to alpine meadows.

The Euro‐Siberian region is characterized by a diverse and complex climate. A transitional climate prevails in the Marmara region, where the influence of the Mediterranean climate diminishes as one moves north. Broad‐leaved forests, including species such as beech (*Fagus* sp.), chestnut ( *Castanea sativa* ), linden (*Tilia* sp.), oaks (*Quercus* sp.), maple (*Acer* sp.), ash trees ( *Fraxinus excelsior* ), junipers (*Juniperus* sp.), alder (*Alnus* sp.), hornbeam ( *Carpinus betulus* ), and poplar trees (*Populus* sp.), are widespread along the Black Sea coast from the Istranca Mountains to the Georgian border. In higher altitudes, the mix of broad‐leaved and coniferous forests is dominated by Turkish pine ( *Pinus brutia* ), black pine ( *Pinus nigra* ), oriental beech (*Fagus orientalis*), oriental spruce (*Picea orientalis*), fir (*Abies nordmanniana*), and Scots pine ( *Pinus sylvestris* ).

The Irano‐Turanian region covers Central and Eastern Anatolia, a region with high endemism (Noroozi et al. 2019) primarily characterized by a continental climate. The region's elevation increases dramatically from west to east, and steppe plant communities dominate in Central Anatolia. Forest cover is lower here compared to the other two regions, consisting primarily of mixed and evergreen coniferous forests. Key tree species include oak (*Quercus* sp.), black pine ( *Pinus nigra* ), Scots pine ( *Pinus sylvestris* ), junipers (*Juniperus* sp.), and hornbeam (*Carpinus* sp.).

### 2.2 Data Collection

**FIGURE 1:**
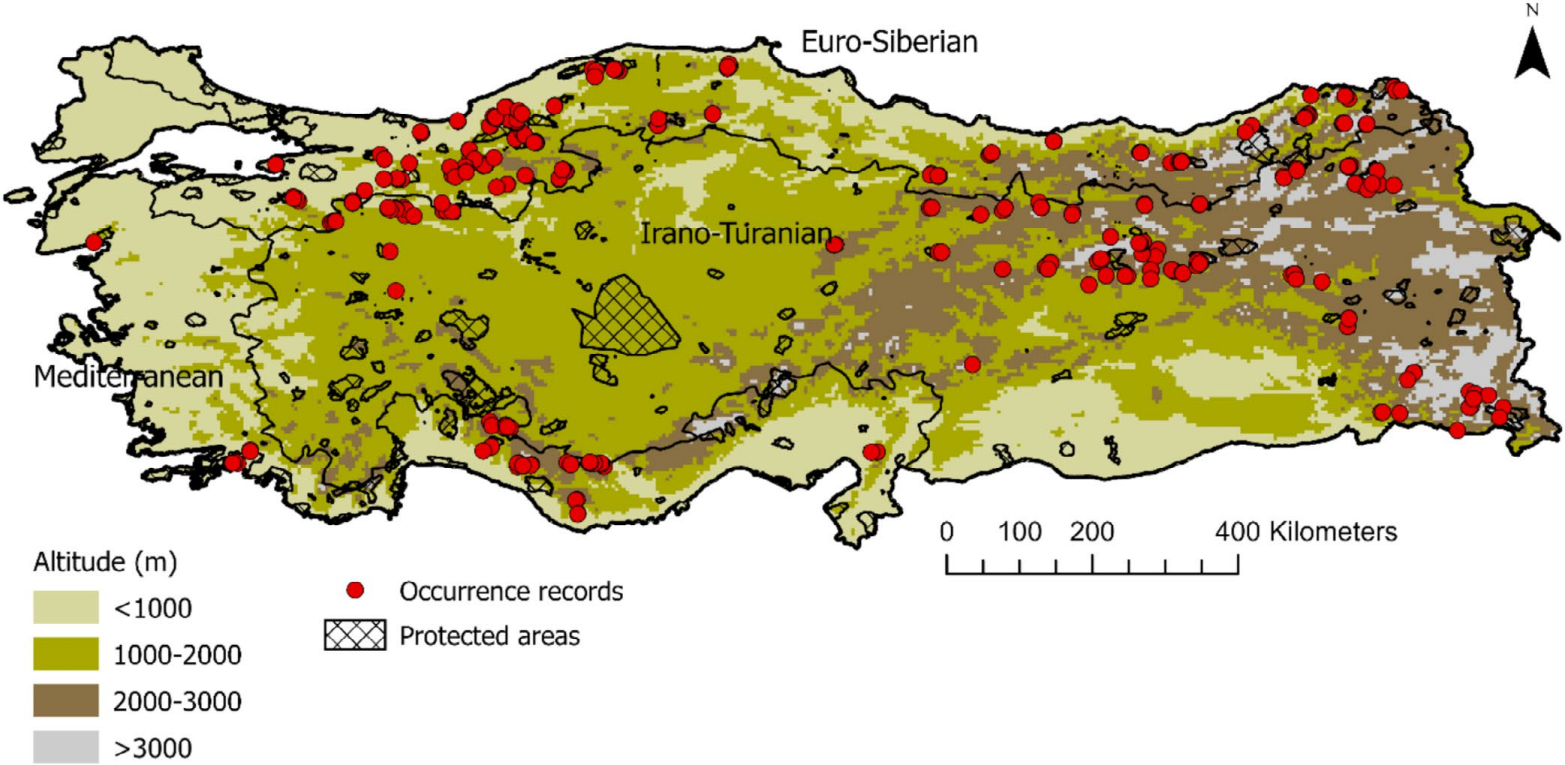
Occurrence records (*n* = 608) and the borders of the biogeographic regions across Türkiye.

Between May 2020 and October 2021, extensive fieldwork was conducted by our research team to gather data on signs of brown bear ( *Ursus arctos* ) presence (Figure S1) across Türkiye. The data collection process involved a systematic random design, recording 608 presence points (Figure 1; see Supporting Information S1). To address potential spatial autocorrelation effects, we utilized the spThin package in R (Aiello‐Lammens et al. 2015) to filter out spatial clustering of points at a 10 km scale across the study area. After spatial thinning, modeling continued with 121 presence records.

### 2.3 Environmental Variables

Our ecological niche modeling incorporated various predictors, including climatic, topographic, and anthropogenic variables (Table S1). We sourced the current 19 bioclimatic layers from the WorldClim database (Fick and Hijmans 2017) with a 2.5 arc‐min resolution (approximately 4.6 km at the equator). Topographic variables, including elevation, slope, aspect, and terrain ruggedness index (TRI), were derived from the elevation data of the Shuttle Radar Topography Mission (SRTM). Anthropogenic factors were also taken into account as distal variables. These included distance to forests, croplands, grasslands, roads, built‐up areas, and water resource predictors, as well as population density and the Global Human Modification of Terrestrial Systems dataset. Detailed information on all the environmental predictors used is provided in the Supporting Information S1.

### 2.4 Multicollinearity

To account for multicollinearity among the spatial variables, data was analysed using R *v4.3.0*. Variables exhibiting a correlation coefficient greater than |0.7| and a variance inflation factor exceeding five were excluded from further analysis (Figure S2). Following this, 17 variables were retained for subsequent modeling, including distance to Forest, Cropland, Built‐Up areas, Road, Water, Slope, Aspect, TRI, Mean diurnal range, Annual mean temperature, Mean temperature of wettest quarter, Temperature seasonality, Mean temperature of driest quarter, Precipitation of driest months, Precipitation of wettest months, Population Density, and Global Human Modification of Terrestrial Systems (Table S1).

### 2.5 Future Forecasting

For future projections, bioclimatic variables with a resolution of 2.5 arc‐minutes were downloaded from WorldClim 2.1, based on the CMIP6 (Coupled Model Intercomparison Project Phase 6) data, as featured in the sixth assessment report (AR6) of the Intergovernmental Panel on Climate Change. Three widely used general circulation models (GCMs); MIROC6, CNRM‐CM6–1, and MPI‐ESM1‐2‐HR, with three Shared Socioeconomic Pathways/Representative Concentration Pathway scenarios, including SSP1/RCP2.6 as optimistic future, SSP3/RCP7.0 as intermediate future, and SSP5/RCP8.5 as pessimistic future, were downloaded for both 2050 (average for 2041–2060) and 2070 (average for 2061–2080). These GCMs were chosen since they had good performance in predicting accurate distribution modeling across Europe in other studies (Canturk and Kulaç 2021; Gür 2022; Naimi et al. 2022).

### 2.6 Habitat Suitability Modeling

To predict the current and future distributions of brown bears in Türkiye, an ensemble forecasting approach was employed using the *biomod2* package (Thuiller et al. 2009). Six modeling algorithms were applied: Generalized Linear Model (GLM), Maxent, Random Forest (RF), Artificial Neural Network (ANN), Generalized Boosted Model (GBM), and Extreme Gradient Boosting Training. A total of 10,000 pseudo‐absence points were generated (Barbet‐Massin et al. 2012), and the dataset was split into training (70%) and testing (30%) (Pant et al. 2021). Three cross‐validation iterations were conducted, and pseudo‐absence points were regenerated to minimize random bias.

Model performance was assessed using the true skill statistic (TSS), and models with TSS scores greater than 0.6 were selected for the ensemble. Predictions were generated using a weighted average approach (Araújo and New 2007; Thuiller et al. 2009). Binary maps were generated by applying a threshold that maximized TSS, and range changes (i.e., gain, loss, stability, or no occupancy) were analyzed (see Supporting Information S3). Habitat range changes were evaluated by averaging future scenarios, and suitable areas were categorized by various altitude ranges (i.e., < 1000; 1000–2000; 2000–3000; > 3000 m). Land‐cover proportions of suitable habitats were analyzed, and the overlap with established protected areas was examined (see Supporting Information S3).

## 3 Results

### 3.1 Current Potential Distribution, Contribution of Variables, and Model Performance

**FIGURE 2:**
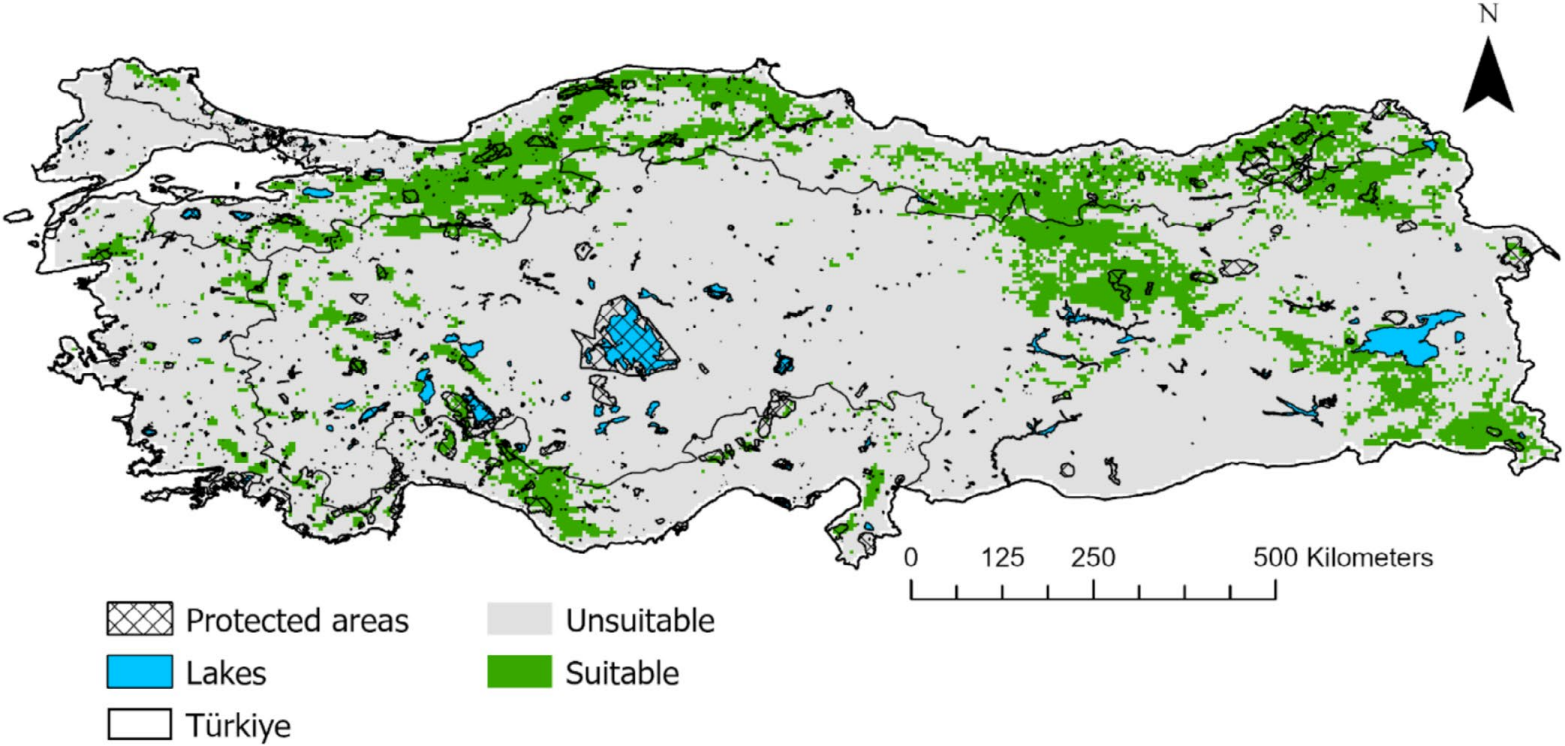
The potential current distribution of brown bears across Türkiye derived from the binary map based on the ensemble model.

The six ecological niche models evaluated in this study showed varying levels of predictive accuracy. The ensemble model demonstrated strong predictive performance (AUC: 0.96, TSS: 0.81) (Figure S3). According to this ensemble model, about 17.3% of Türkiye's land area, equivalent to 135,556 km^2^, is currently suitable for brown bear habitation (Figure 2). The most suitable habitats are located in the Euro‐Siberian (46%, 62,356 km^2^), Irano‐Turanian (43%, 58,289 km^2^), and Mediterranean (11%, 14,911 km^2^) biogeographic regions (Figure 2).

**FIGURE 3:**
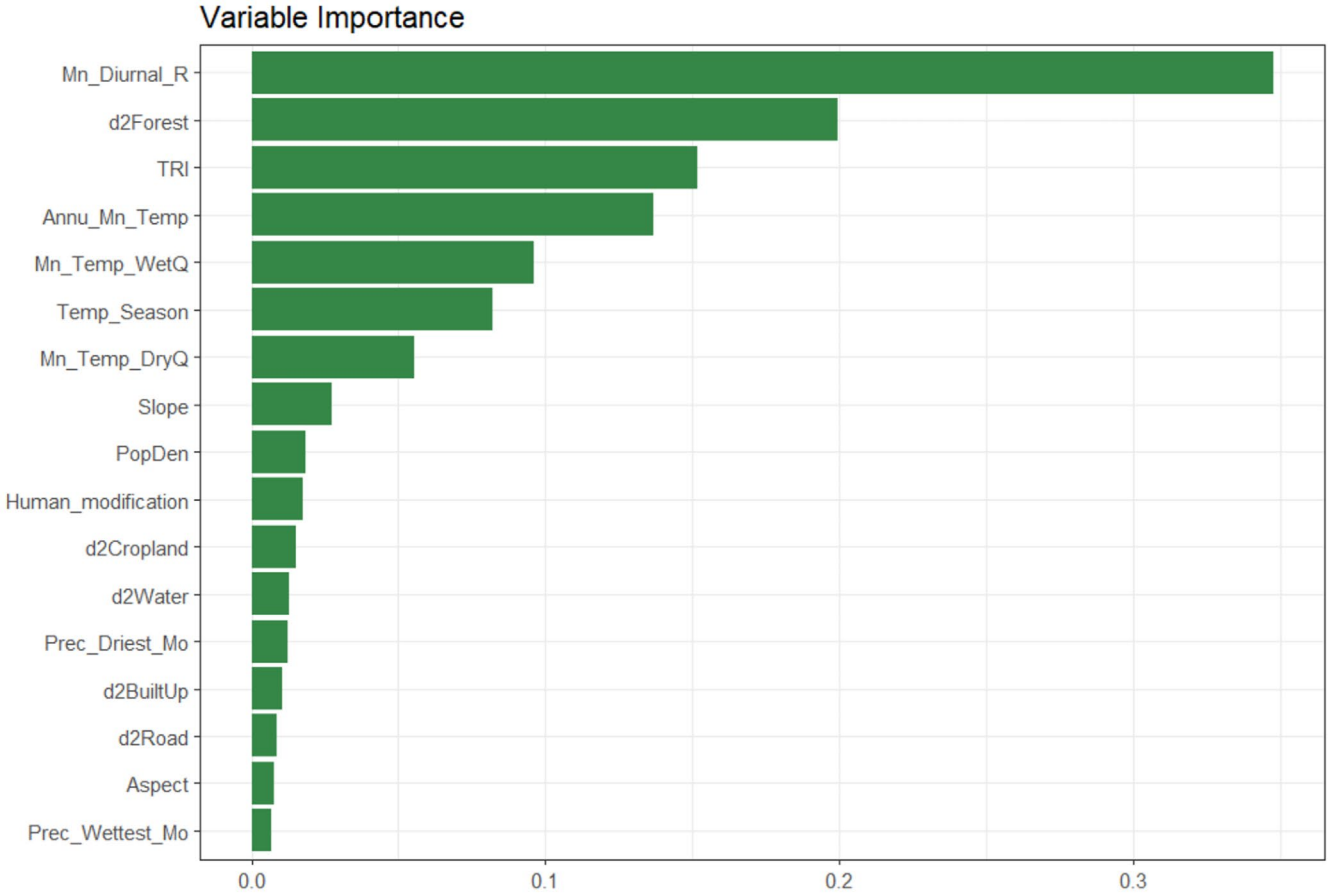
Percent contribution of environmental predictors to the final ensemble model for ecological niche modeling of brown bears across Türkiye.

**FIGURE 4:**
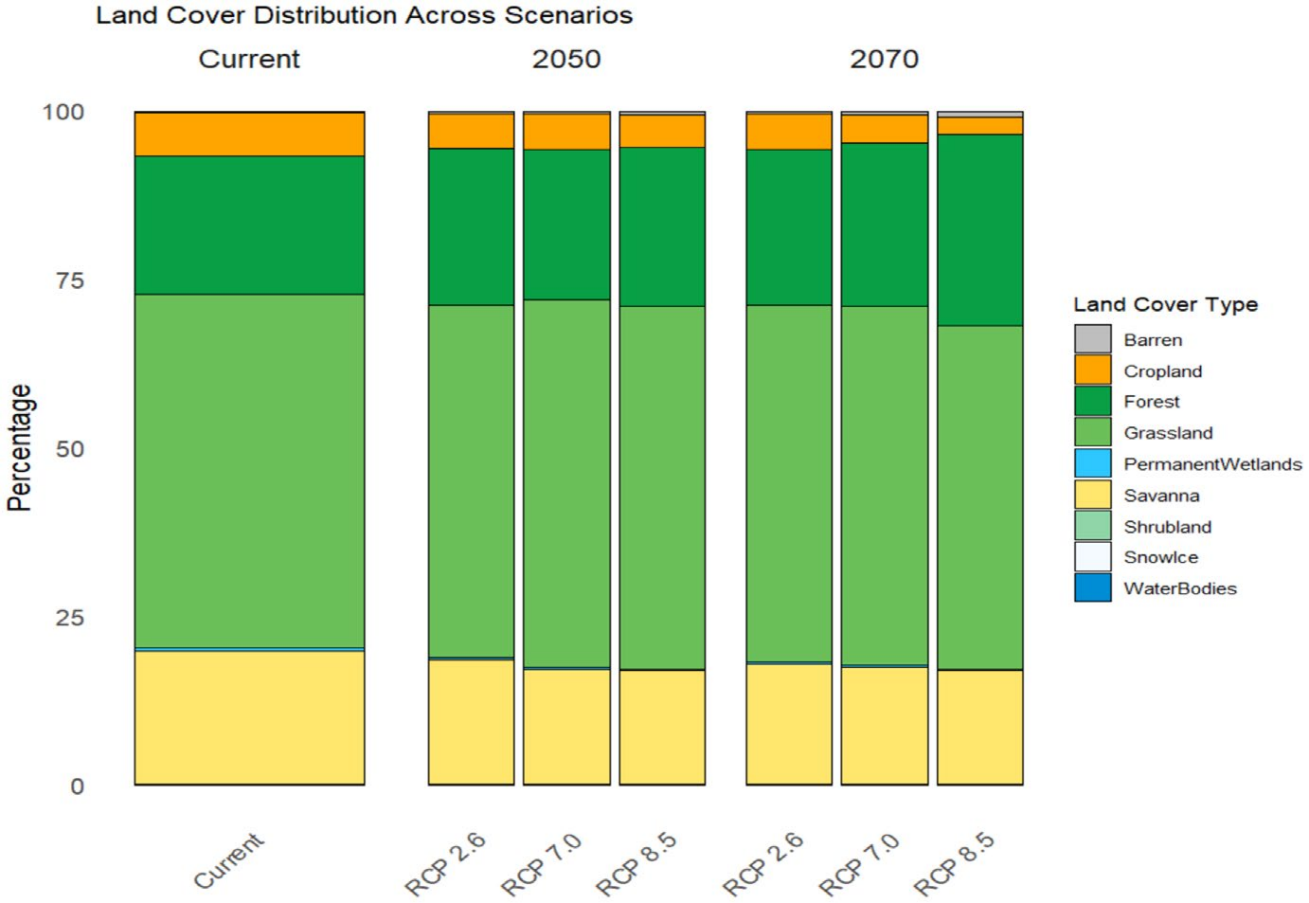
Land‐cover distribution of suitable patches based on average rasters of three GCMs (MIROC6, CNRM‐CM6‐1, MPI‐ESM1‐2‐HR) across scenarios as well as present day.

The contribution of environmental variables varied across the models. The Mean diurnal range was the most significant, accounting for 35% of the influence, followed by Distance to the forest (20%), TRI (15%), and Annual mean temperature (14%) (Figure 3). Brown bear presence probability decreased as the Mean diurnal range and Precipitation of driest months increased, along with certain anthropogenic factors such as proximity to built‐up areas and roads (Figure S4). However, most anthropogenic predictors, except for Distance to forest, had less impact (< 5%), indicating that bioclimatic variables largely influence model behaviors (Figure 3). Based on the MODIS land‐cover dataset, the current suitable habitats for brown bears predominantly consist of Grassland (52.3%), Forest (20.6%), Savanna (19.7%), and Cropland (6.3%) (Figure 4), highlighting the species' significant presence in landscapes modified by human activities.

### 3.2 Future Habitat Suitability of Brown Bears Across Türkiye

**FIGURE 5:**
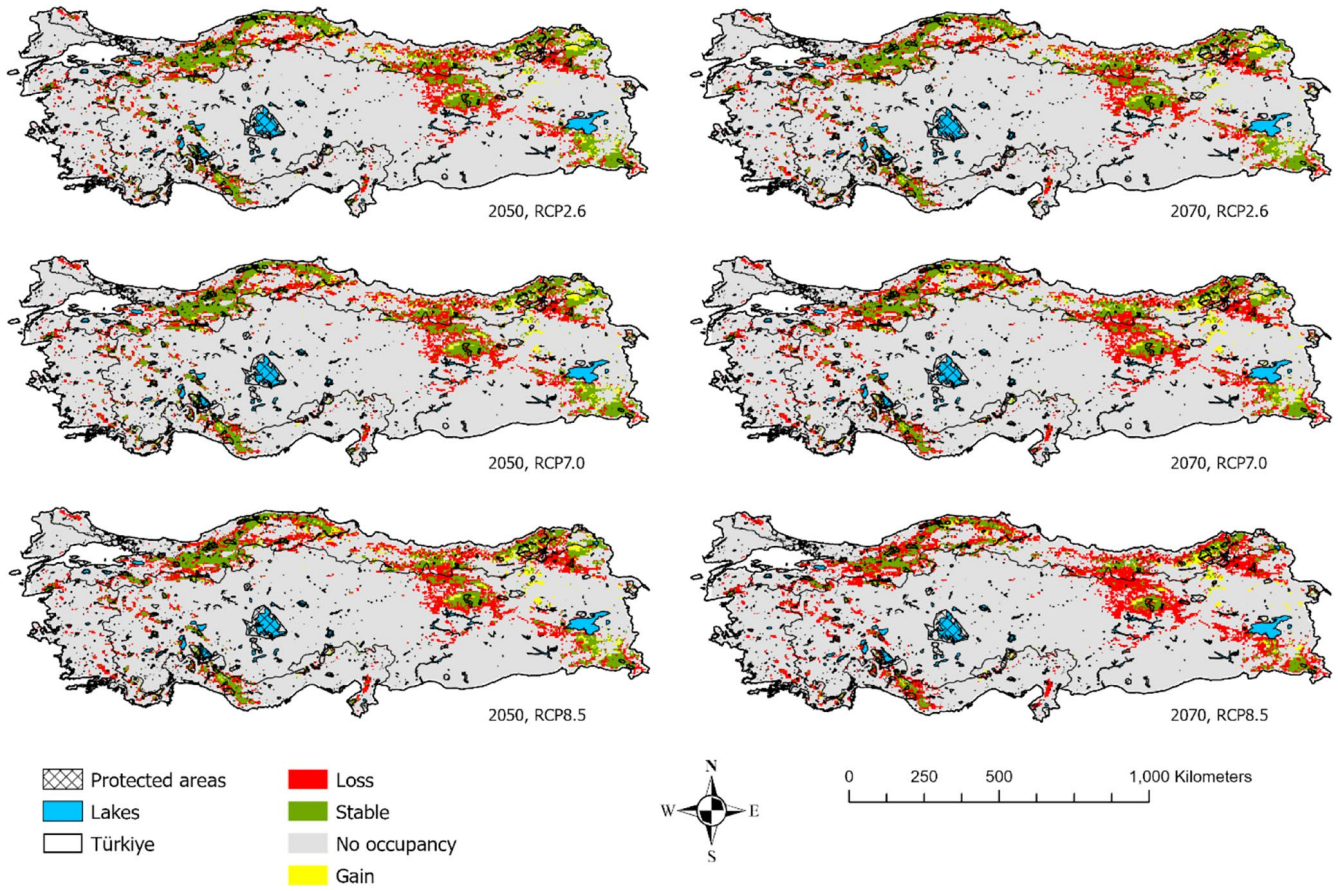
Ensemble forecasting of the future range change by considering optimistic (RCP 2.6), intermediate (RCP 7.0), and pessimistic (RCP 8.5) scenarios for brown bears of Türkiye in both 2050 and 2070 based on the average of three GCMs (MIROC6, CNRM‐CM6‐1, MPI‐ESM1‐2‐HR).

Our results indicate that the suitable habitats for brown bears are highly vulnerable to the impacts of climate change (Figure 5). Among the GCMs, MIROC6, CNRM‐CM6‐1, and MPI‐ESM1‐2‐HR showed the highest sensitivity in predicting brown bear habitat suitability for 2050 and 2070 (Figure S5). Under an optimistic climate scenario, habitat loss by 2050 ranged from 3% (MPI‐ESM1‐2‐HR) to 57% (MIROC6), while under a pessimistic scenario, losses were projected between 17% (MPI‐ESM1‐2‐HR) and 64% (MIROC6) (Table S2). By 2070, the situation worsened, with habitat losses ranging from 37% (MPI‐ESM1‐2‐HR) to 81% (MIROC6) in the pessimistic scenario (Table S2). Although some habitat gain is predicted, the overall trend suggests a significant contraction across all GCMs and climate scenarios (Figure S6). In summary, brown bear habitats in Türkiye are projected to decrease by 39%–47% by 2050, and by 2070, this contraction could reach 40%–65%, depending on the scenario (Figure S6).

Future projections suggest that habitat loss areas for brown bears will predominantly occur in the Mediterranean and Irano‐Turanian regions across the three GCMs for both 2050 and 2070 (Figure S7). For 2050, the Euro‐Siberian region is projected to lose 33%–43% of its suitable habitats, the Irano‐Turanian region 44%–51%, and the Mediterranean region 51%–59%, depending on the scenario (Figure S7). By 2070, these losses are expected to worsen, with the Euro‐Siberian region projected to lose 34%–57%, the Irano‐Turanian region 43%–74%, and the Mediterranean region 53%–77% of suitable habitats (Figure S7). This indicates that significant portions of brown bear habitats, especially in the Mediterranean and Irano‐Turanian regions, are at high risk of disappearing in the coming decades.

### 3.3 Potential Habitat Suitability, Altitudinal and Latitudinal Range Shifts

**FIGURE 6:**
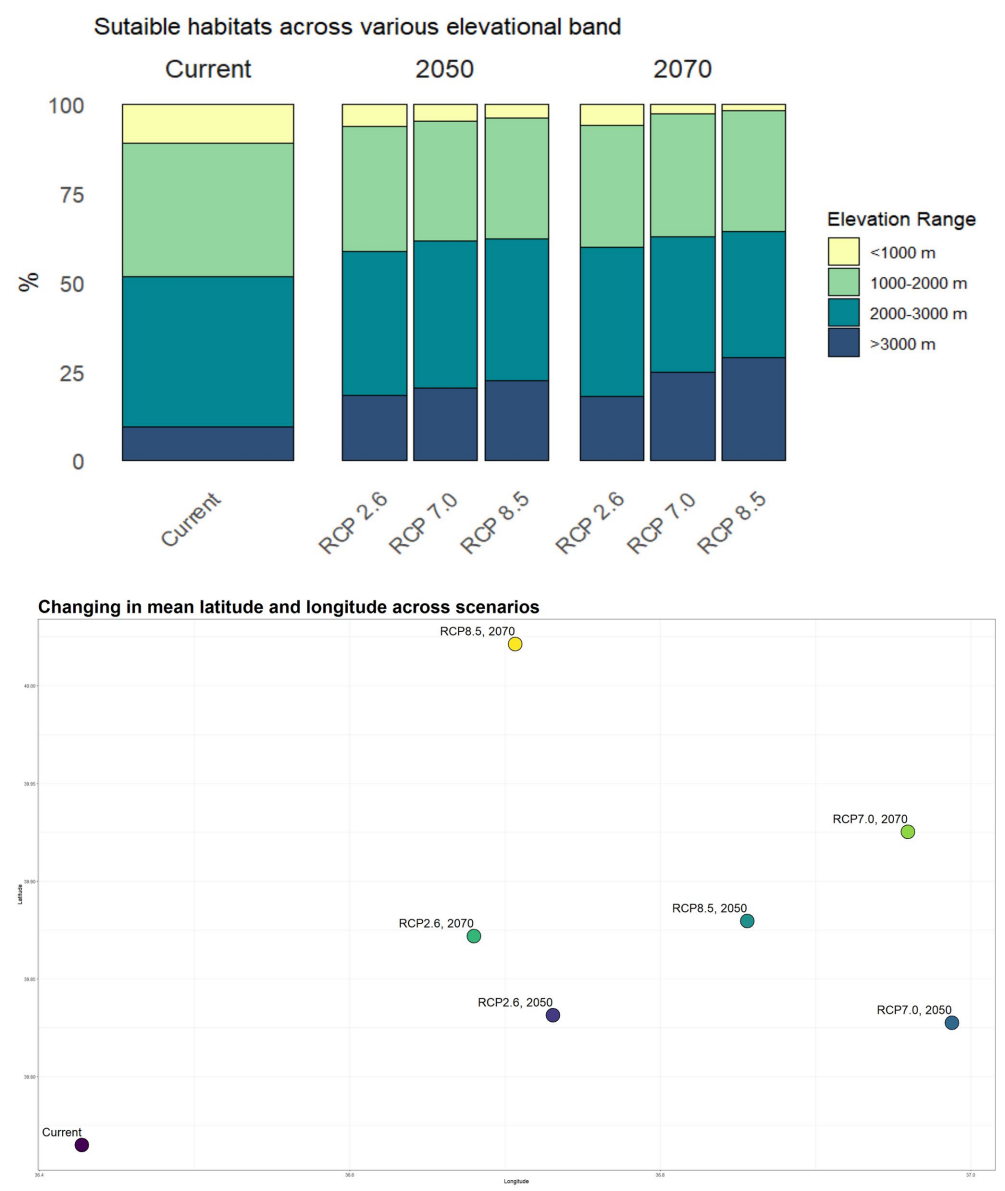
(A; top) Suitable habitats across various elevational zone, which is based on average rasters of three GCMs (MIROC6, CNRM‐CM6‐1, MPI‐ESM1‐2‐HR). (B; bottom) The spatial variation of the suitable range of brown bear across Türkiye by considering current and future scenarios.

Our model reveals a notable correlation between elevation and the distribution of suitable habitats for brown bears. Currently, approximately 10% of the suitable habitats (13,556 km^2^) are located below 1000 m, 37% (50,156 km^2^) between 1000 and 2000 m, 42% (56,934 km^2^) between 2000 and 3000 m, and 10% (13,556 km^2^) above 3000 m (Figure 6A). However, climate change is predicted to cause a redistribution of suitable habitats, with a noticeable shift toward higher elevations (Figure 6A). The average altitude of brown bears' suitable patches for present day in Türkiye is 1931 m. Under future climate change scenarios, the average altitude is projected to range between 2098 and 2176 m by 2050, and between 2108 and 2266 m by 2070 (Table S3). Furthermore, this shift is not limited to elevation but also reflects a latitudinal displacement, indicating that brown bear habitats are expected to move northward as a direct response to climate impacts (Figure 6B). Additionally, the west‐to‐east increase in elevation across Türkiye supports the model's prediction that suitable habitats will shift both longitudinally and to higher elevations. This suggests that in the near future, brown bears will find more favorable conditions at higher altitudes, especially in the country's eastern regions (Figure 6B). This elevational and latitudinal shift highlights the pressing need for conservation strategies that account for these geographical and environmental changes.

### 3.4 Potential Habitat Suitability and Established Protected Areas

**FIGURE 7:**
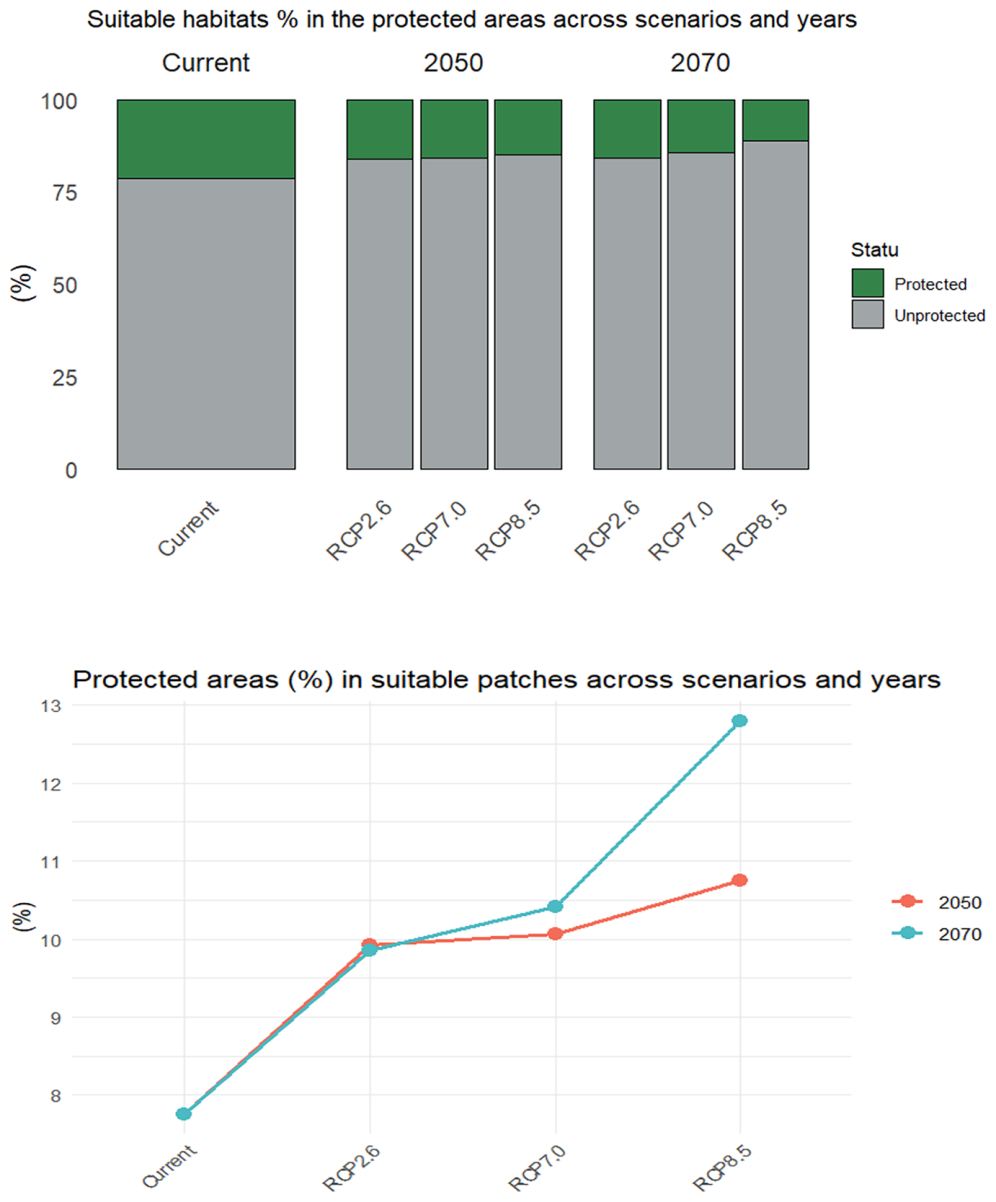
(A; top) Suitable habitat proportion in the selected protected areas across scenarios and years. (B; bottom) Selected protected areas proportion in the suitable patches across scenarios and years.

Currently, 21.4% (29,009 km^2^) of the suitable habitats for brown bears fall within Türkiye's protected areas (Figure 7A). However, this proportion is expected to decline under future climate scenarios, dropping to 15.0%–16.1% by 2050 and 11.3%–15.9% by 2070 (Figure 7A). This indicates a significant reduction in the overlap between protected areas and suitable bear habitats, which could exacerbate conservation challenges.

## 4 Discussion

Our ensemble model estimated that approximately 135,556 km^2^ (17.3%) of Türkiye's land area is currently suitable for brown bear habitation. This amount is slightly lower than the estimates from previous studies, such as those by Ambarlı et al. (2016) and McLellan et al. (2017), which suggested that brown bears could occupy 20%–30% of the country's land area, primarily concentrated in the northern and eastern regions. Both of these studies highlighted that southern habitats tend to be fragmented, consistent with our findings. Furthermore, our model identified the Euro‐Siberian, Irano‐Turanian, and Mediterranean biogeographic regions as the most suitable areas for brown bears, mirroring the conclusions of earlier research (Ambarlı et al. 2016; McLellan et al. 2017). These regions continue to provide crucial habitats for brown bears despite the ongoing challenges posed by climate change and habitat degradation.

Mean diurnal range (35%), Distance to forests (20%), and TRI (15%) are the most influential variables affecting brown bear presence probability in the country. Other temperature‐related variables, including Annual mean temperature (~14%), Mean temperature of the wettest quarter (~10%), Temperature seasonality (8%), and Mean temperature of the driest quarter (~6%), also emerged as significant predictors. Based on the response curves (Figure S4), the likelihood of brown bear presence peaks within the mean diurnal range of 8°C–10°C. The mean diurnal range also plays a significant role in shaping brown bear distribution across various landscapes (Penteriani et al. 2019; Hosseini et al. 2021; Ashrafzadeh et al. 2022). For instance, a study conducted in the Zagros Mountains estimated that the most suitable habitats for brown bears are located in regions where the mean diurnal temperature range is below 11.5°C (Hosseini et al. 2021). Similarly, in the Cantabrian Mountains, the mean diurnal range has been identified as a critical predictor influencing the distribution of both brown bears and their food resources such as blueberry, beech, and chestnut (Penteriani et al. 2019). Brown bears were found to prefer specific temperature ranges, underlining the role of climate in shaping their distribution. For example, our model indicates that the current suitable patches for brown bears will face an average annual temperature increase of 2.0°C–2.4°C by 2050 and 3.3°C–4.3°C by 2070, depending on the scenario (Figure S8). Indeed, as a result, while many of the suitable habitats of brown bears for present day will disappear shortly, even in the case of a habitat shift, the species will still face an average annual temperature increase ranging from 1.1°C to 1.3°C by 2050 and from 1.8°C to 2.4°C by 2070 (Figure S8).

Contribution of Distance to forest highlights the species' dependence on forested areas for essential activities such as cover, foraging, and denning (Recio et al. 2021; Ziółkowska et al. 2016; Naderi et al. 2024). The parabolic relationship between distance to forests and bear presence (Figure S4) suggests that brown bears favor proximity to forests or grasslands, emphasizing the importance of preserving and connecting these productive landscapes for effective conservation. Suel (2019) reported that natural open areas and forests represent important habitats for brown bears in the Mediterranean. TRI is a commonly used environmental variable for measuring habitat selection by brown bears (Suel 2019; Ashrafzadeh et al. 2022, 2023; Dilts et al. 2023). As a general pattern, brown bears tend to select more rugged or higher‐altitude areas to avoid human impact (Goldstein et al. 2010; Peters et al. 2015; Piédallu et al. 2019; Suel 2019; Zarzo‐Arias et al. 2019). These regions offer low‐risk habitats with shelter, but resource availability remains a key factor. Although brown bears increasingly select anthropogenic habitats due to accessible food resources, such areas are crucial for fulfilling their ecological needs, especially for hibernation (Faure et al. 2020). However, increasing road access (Bilici and Akay 2021) to previously undisturbed areas in Türkiye has raised concerns about habitat fragmentation and bear mortality. Studies indicate that road density, especially in the Western Black Sea region where bear populations are high (Dagtekin et al. 2024), can influence bear circadian behaviors (Ordiz et al. 2014) and act as barriers to habitat connectivity (Epps and Keyghobadi 2015). Increased human presence and road density are correlated with higher mortality risks for brown bears (Parsons et al. 2023), and the pursuit of anthropogenic food sources such as garbage dumps can alter bears' seasonal habitat use and have demographic consequences (Kemahlı et al. 2023; Dagtekin et al. 2024).

The results of our future habitat suitability projections indicate that brown bears in Türkiye face substantial challenges due to climate change. Under various climate change scenarios, brown bear habitats are predicted to contract substantially, particularly in the Mediterranean and Irano‐Turanian regions, which currently support some of Türkiye's important brown bear populations. By 2070, under the pessimistic scenario, habitat loss is expected to range from 40% to 66% (Figure S6). However, this average loss is projected to be more severe in the Mediterranean region, ranging from 53% to 77%, and in the Irano‐Turanian region, ranging from 43% to 74% (Figure S7). Notably, the limited potential for range expansion suggests that brown bears may struggle to find new suitable habitats to offset these losses. This raises concerns about their long‐term survival in Türkiye. Our model projects that brown bears in Türkiye will face warmer temperatures in the future (Figure S8), which may affect key behaviors such as denning. For example, ambient temperature is a crucial factor in regulating the timing of den entry and exit for brown bears (Evans et al. 2016), and climate change is expected to influence their denning patterns (González‐Bernardo et al. 2020). In the Mediterranean region, warmer temperatures and reduced snow cover may prevent some brown bears from hibernating annually (Soyumert et al. 2020). Furthermore, wildfires pose a significant threat to the core habitats of brown bears and could have more profound impacts on current and future bear distributions than initially anticipated (Khosravi et al. 2022). Human land use across Anatolia is also expected to have a more dramatic impact on biodiversity than previously assumed in the near future (García‐Vega and Newbold 2020; Gür 2022). The southernmost population of brown bears globally resides in the Turkish pine forests of the western Mediterranean (Soyumert et al. 2020). However, our model indicates that most of these habitat patches are likely to become unsuitable in the near future. In Türkiye, brown bears predominantly consume plant‐based diets rather than livestock (Ambarlı 2016), but climate change is expected to disrupt their food resources by shifting the availability and reducing the quantity of key food sources (Penteriani et al. 2019; Qin et al. 2020; Pérez‐Girón et al. 2024). As climate change alters the ripening times of fruits that bears rely on during their hyperphagic period, they may be forced to seek alternative anthropogenic food sources (Pérez‐Girón et al. 2022). This shift in foraging behavior is likely to intensify human–wildlife conflicts, particularly in areas with scattered settlements, such as the Euro‐Siberian region, where incidents like crop damage, house break‐ins, and damage to beehives are predicted to increase (Ambarlı and Bilgin 2008; Dai and Hacker 2021). In the Irano‐Turanian region, on the other hand, livestock predation is expected to rise (Kusak and Şekercioğlu 2021; Sıkdokur et al. 2024). Bears' foraging patterns are also likely to involve larger seasonal movements in search of food, which could expose them to additional risks (Penteriani et al. 2019). Brown bears play a critical role in ecosystems by dispersing seeds over long distances, supporting plant community regeneration (García‐Rodríguez et al. 2021). However, this reciprocal relationship between bears and their food sources may be disrupted by climate change (Pérez‐Girón et al. 2024). In Türkiye, human‐driven factors like deforestation or land conversion could exacerbate this disruption. Projections show that future brown bear habitats may encompass a larger proportion of forest cover, which may influence the availability of food resources (Figure 4). For instance, the broad‐leaved forest species Oriental beech (*Fagus orientalis*), a crucial resource for brown bears, is projected to shift northward and experience a significant range contraction in the future (Dagtekin et al. 2020; Ayan et al. 2022). Similar patterns have been observed for the European hornbeam ( *Carpinus betulus* ), another important component of Türkiye's forests (Varol et al. 2022). Brown bears have developed certain adaptive behaviors to cope with forest changes, such as using young and aged forest patches to avoid overheating, but these adaptations vary between genders (Stewart et al. 2013). However, as forest degradation continues and habitat quality diminishes, brown bears may face additional stresses, including higher risks of overheating and increased infanticide (Nellemann et al. 2007).

Elevation‐based analysis suggests that climate change will impact brown bear habitat suitability at various elevations, requiring brown bears to adapt to shifting elevation zones. According to the results, suitable habitats for brown bears in Türkiye are projected to shift to higher altitudes by 167–245 m by 2050 and by 177–335 m by 2070, depending on the scenario (Table S3). While brown bears are typically a high‐altitude species, their habitat preferences can also be influenced by human land use and changes in resource availability (Lamamy et al. 2019; Penteriani et al. 2019; Schrag et al. 2008). In Türkiye, transhumance, a traditional practice of seasonal livestock movement, is common at higher altitudes, which places virgin habitats above 2000 m at risk from increased human activity. Due to changing climate, brown bears are expected to shift to higher altitudes in other regions as well (Dai and Peng 2021; Baral et al. 2024). The predicted northward and altitudinal shifts of suitable habitats imply that brown bears will face additional pressures in adapting to these new conditions. For instance, the absence of food sources like termites and ants at higher elevations could impact the energy budget of brown bears (Baral et al. 2024). As elevation increases, the geographical area and, consequently, the available habitat space decreases for species (Sekercioglu et al. 2008). Considering the potential shift in floristic composition, intraspecific and interspecific competition for limited food resources may increase (Penteriani et al. 2019; Dagtekin et al. 2020; Zahoor et al. 2021; Ayan et al. 2022). Additionally, due to these diminishing resources, livestock‐related conflicts may pose increasing threats to brown bears, particularly in high‐altitude regions. Such fluctuations underscore the importance of maintaining landscape connectivity to facilitate the movement of brown bears across different elevation zones and between core habitat patches.

Protected areas are essential for conserving biodiversity and providing a safe refuge for species facing habitat loss (Atmiş et al., 2018). Globally, the number of PAs has increased, with 16.4% of Earth's terrestrial surface currently under protection (UNEP‐WCMC, 2020). However, only 7% of Türkiye's total area is protected, and a mere 7% of its terrestrial regions hold this status (NCNP 2022). As ecological processes are dynamic and influenced by both spatial and temporal factors, PAs' current status and effectiveness need to be reevaluated in the context of climate change and shifting land‐use patterns. Türkiye's PAs are already under significant anthropogenic pressures, threatening their conservation role (Şekercioglu et al., 2011; Atmiş 2018). For instance, Sıkdokur et al. (2024) stated that the protected areas in Türkiye are hotspots for human–brown bear conflicts and that human activities in these areas, which harbor the core habitats of bears, should be reduced. Similar conservation challenges have been estimated in other regions (Mukherjee et al. 2021; Su et al. 2018). Unfortunately, PA governance in Türkiye is legally and administratively complex, with overlapping protection statuses and varying levels of restrictions depending on the type of PA (Birben 2020; Atmiş 2018). These complexities bring some governance and cooperation issues for PA management as well as harmony issues with IUCN criteria (Atmiş 2018). While minimizing human impacts on PAs is essential, conservation‐oriented PAs in Türkiye have significantly decreased, while utilization‐focused PAs have expanded (Atmiş 2018). Besides, our model highlights that forested areas and open landscapes are critical habitats for brown bears, accounting for approximately 73% of suitable habitats. While 8% of the world's forests are designated as PAs (Schmitt et al. 2009), this proportion is even lower in Türkiye's forests (Birben 2020). This low percentage, when considered alongside the increase in non‐forestry land uses such as mining, energy development, and recreational activities (GDF 2022), deepens the conservation crisis for wildlife and pristine areas. According to findings, despite some potential increases in the proportion of protected areas within suitable patches in future scenarios (Figure 7B), the overall percentage of suitable habitat that is protected is expected to decrease (Figure 7A). These outputs underscore that the established PAs are currently insufficient in terms of suitable bear habitats and are far from improving their response to climate change. On the other hand, the expected increase in the proportion of PAs within suitable habitats in the near future means that enhancing the effectiveness of established PAs will strengthen their buffer role against the challenges brown bears are likely to face. In this context, performing connectivity modeling as a next step to make PAs support the ecological network of brown bears will be a critical implication. Ultimately, maximizing the conservation function of established PAs through conflict mitigation strategies, expanding their boundaries beyond symbolic surfaces, and designing new PAs that prioritize conservation functions that enhance habitat connectivity will provide a dynamic conservation framework.

The threat to brown bear habitats extends beyond Türkiye, with significant losses projected across different regions of the species' range. In Central Asia, for example, suitable brown bear habitats within protected areas are expected to decrease by 1,103,912 km^2^ shortly (Su et al. 2018). Similarly, in the Hindu Kush Himalayan region, the suitable habitats for *Ursus arctos pruinosus* and *Ursus arctos isabellinus* are forecasted to decline significantly and shift to higher altitudes due to climate change (Dai and Peng 2021). In Iran's Chaharmahal and Bakhtiari regions, a pessimistic scenario predicts up to a 45% loss of brown bear habitats by 2050 (Ashrafzadeh et al. 2022). Even more alarmingly, habitat losses of up to 91% are predicted for brown bears in Sanjiangyuan National Park, China, by 2070 (Dai et al. 2019). These projections highlight the growing vulnerability of brown bears to climate change and emphasize the urgent need for coordinated, transboundary conservation efforts (Almasieh et al. 2019; Kopatz et al. 2021; Reljic et al. 2018). In this context and based on our findings, maintaining habitat connectivity along transboundary regions, particularly toward Iran and the Caucasus, is essential.

### 4.1 Study Limitations

Understanding the habitat vulnerability of brown bears provides a baseline for assessing their responses to future climate and land use and land‐cover (LULC) changes. However, the anthropogenic predictors in our study were not included in future projections, which could lead to underestimating dramatic changes in some cases. Consequently, our findings may present a conservative outlook. Unlike GCMs, LULC projections carry significantly more uncertainty. Similar to prior studies (Ashrafzadeh et al. 2019, 2022; Deb et al. 2019; Zarzo‐Arias et al. 2019; Morovati et al. 2020; Mukherjee et al. 2021), we used static anthropogenic predictors. Nevertheless, as highlighted earlier, combining static variables with high driving potential alongside dynamic climatic variables yields more accurate results than excluding them entirely (Stanton et al. 2012). In this model, biotic interactions were not considered. Vegetation covers may change drastically due to climate change (De Frenne et al. 2021), and plant species and the natural prey of bears may also exhibit range shifts (Ali et al. 2021; Khosravi et al. 2021). Therefore, joint effects of biotic interactions should also be considered in projections as future suggestions (Lucas et al. 2023). Considering the dramatic increase in extreme weather events since the 2000s (Walsh et al. 2020), another limitation of this study is the temporal mismatch between bioclimatic variables covering the 1970–2000 time range (Fick and Hijmans 2017) and occurrence records from after 2020 (Gür 2022). Although ENMs provide a valuable approach for understanding species' responses to climate change, they assume that occurrence records represent all populations and that species are in equilibrium with their environment. However, they do not account for any mechanistic relationships or consider local adaptation (Gür 2022). Therefore, in addition to studies examining the ecophysiological responses of brown bears in the near future, there is a need for research that also considers local adaptation, which requires accounting for seasonal and spatial variation. The seasonal variation in the presence points was not considered in this model. For instance, in Türkiye, brown bears typically enter hibernation by late December, depending on the altitude, and emerge from their dens in mid‐spring for cub rearing or mating. In this context, if we divide our collection times of the records into three distinct periods (i.e., Denning [January 1—April 15], Mating [April 16—June 30], and Hyperphagia [July 1—December 31]); 7% (*n* = 44) of the presence points were obtained during denning period, 26.3% (*n* = 160) were collected during the mating period, and 66.4% (*n* = 404) were obtained during the hyperphagia period. Both the bears' habitat selection and the human dominance in different habitat characteristics might be dramatic across the mentioned periods (De Angelis et al. 2021; Bogdanović et al. 2021; Donatelli et al. 2022; Falcinelli et al. 2024; Dagtekin et al. 2024); Black Sea and Eastern Anatolia regions are thought to host 2000–3000 bears, while the Mediterranean region supports only 200–300 individuals (Ambarlı et al. 2016). In this study, ~40% (*n* = 242) of the presence points were obtained from the Euro‐Siberian, ~48% (*n* = 289) from the Irano‐Turanian, and ~13% (*n* = 77) from the Mediterranean basin. Although the occurrence records collected in this study are relatively well‐distributed, more detailed surveys particularly across Eastern Anatolia are essential to gather a higher number of well‐covered occurrence points, which are critical for improving predictions. Besides, this model does not have a gender‐specific interpretation; however, it has been previously stated that the dispersal ranges and habitat uses of bears include gender‐specific outcomes (García‐Sánchez et al. 2022; Bogdanović et al. 2021; Falcinelli et al. 2024), and distribution models can lead to overestimated results for females when genders are pooled together (Gantchoff et al. 2019). Lastly, our models were carried out at the coarse scale; however, fine‐scale studies are essential to understand the effects of climate change and anthropogenic interactions and to improve landscape‐specific conservation strategies.

## 5 Conclusion

Our study provides valuable insights into the current distribution and future vulnerability of brown bear habitats in Türkiye in the face of climate change. The results emphasize the importance of proactive conservation measures, including habitat protection, restoration, and climate adaptation strategies, to ensure the long‐term survival of brown bear populations in the region. Additionally, our findings can inform conservation planning and policy development to mitigate the impacts of climate change on this iconic species.

## Author Contributions

**Ercan Sıkdokur:** conceptualization (equal), data curation (lead), formal analysis (lead), investigation (lead), methodology (lead), visualization (lead), writing – original draft (lead), writing – review and editing (lead). **İsmail K. Sağlam:** conceptualization (equal), formal analysis (equal), investigation (equal), methodology (equal), project administration (equal), writing – original draft (equal). **Çağan H. Şekercioğlu:** conceptualization (equal), data curation (equal), formal analysis (equal), methodology (equal), wiriting – review and editing (equal), project administration (equal). **Irfan Kandemir:** methodology (equal), writing – review and editing (equal). **Ali Onur Sayar:** methodology (equal), writing – original draft (equal). **Morteza Naderi:** conceptualization (lead), data curation (lead), formal analysis (lead), funding acquisition (lead), investigation (lead), methodology (lead), project administration (lead), resources (equal), supervision (lead), validation (lead), visualization (lead), writing – original draft (lead), writing – review and editing (lead).

## Conflicts of Interest

The authors declare no conflicts of interest.

## Supporting information


Data S1:

## Data Availability

All data for this study, including environmental predictors, sample collection information, and model optimization parameters, can be found in the manuscript and Supporting Information S1. Raw data supporting the results of this study are available upon reasonable request. The data that support the findings of this study are openly available at http://doi.org/10.5281/zenodo.13952622 and https://zenodo.org/records/13952622.
