# Supplementary material for: Climate‐Driven Range Shifts and Conservation Challenges for Brown Bears in Türkiye
Source: Ecol Evol. 2025 Apr 1;15(4):e71019. doi: 10.1002/ece3.71019 (PMC11962206; doi:10.1002/ece3.71019)
Supplement: Supplementary file 1 — Data S1: [file ECE3-15-e71019-s001.docx]

**Supplementary Materials**

**Climate-Driven Range Shifts and Conservation Challenges for Brown Bears in Türkiye**

**Ercan Sıkdokur^1^, İsmail K. Sağlam^1^, Çağan H. Şekercioğlu^1,2^, Irfan Kandemir^3^, Ali Onur Sayar^4^, Morteza Naderi^*5^**

^1^Department of Molecular Biology and Genetics, Koç University, Istanbul, Türkiye

^2^School of Biological Sciences, University of Utah, Salt Lake City, UT, US

^3^Department of Biology, Ankara University, 06100, Ankara, Türkiye

^4^Department of Forestry, Çankırı Karatekin University, 18200, Çankırı, Türkiye

^5^Department of Biology, Faculty Sciences, Sakarya University, Sakarya, Türkiye

**^*^Corresponding author:** mortezanaderi@sakarya.edu.tr

**S1. Data collection and environmental predictors**

Between May 2020 and October 2021, extensive fieldwork was conducted by our research team to gather data on signs of brown bear (*Ursus arctos*) presence across Türkiye. The data collection process involved a systematic random design, recording 608 presence points (242 from Euro-Siberian; 289 from Irano-Turanian; 77 from Mediterranean regions) (Fig. 1). The presence points were determined based on various indicators such as footprints (26), rubbing tree signs (13), hair & tissue samples (15), and scat samples (554) (Fig. S1). We employed the ArcGIS Survey123 application (ESRI, USA) to record the precise geographical coordinates of the presence points. To mitigate potential spatial autocorrelation effects, we used *spThin* package in R (Aiello-Lammens et al., 2015) to filter the spatial clustering of points at the 10 km scale across the study area. .

Our ecological niche modeling incorporated various predictors including climatic, topographic, and anthropogenic variables (Table S1). We sourced the current 19 bioclimatic layers from the WorldClim database (Fick & Hijmans, 2017) with 2.5 arc-min resolution (approximately 4.6 km at the equator). Topographic variables, including elevation, slope, aspect, and terrain ruggedness index (TRI), were derived from the elevation data of the Shuttle Radar Topography Mission (SRTM).

Anthropogenic factors were also taken into account as distal variables. These included distances to forests, croplands, grasslands, roads, built-up areas, and water resources, as well as population density and the Global Human Modification of Terrestrial Systems. Forest, Cropland, Grassland and Built-Up layers were extracted from the MODIS/Terra+Aqua Land Cover Type Yearly L3 Global 500 m SIN Grid database (Sulla-Menashe & Friedl, 2018). Road network data were retrieved from the ArcGIS World Roads layer, with attributes called major roads and highways. Data on water sources (1^st^-4^th^ river order attribute) were extracted from the HydroRIVERS database for Türkiye (Lehner & Grill, 2013). Proximity to these variables was calculated using the Euclidean Distance tool in ArcGIS. Population Density data were downloaded from the Global Human Settlement Layer database for 2021 (Schiavina et al., 2023). Global Human Modification of Terrestrial Systems data was obtained from the NASA EarthData (Kennedy et al., 2020).

Environmental variables that have high potential to drive brown bear distribution to improve ecological niche modeling were selected by considering the literature. For instance, roads, human settlements, and farmlands can represent important ecological traps and affect brown bears’ foraging behavior as well as population persistence (Lamb et al., 2017; Parsons et al., 2023; Roever et al., 2010). Villages attract bears due to the presence of orchards, crops, and garbage dumps in their vicinity. Orchards and accessible crops readily attract bears, potentially converting agricultural fields into ecological traps. When space and food availability are restricted in natural habitats, wide-ranging mammals like bears may shift towards human-dominated landscapes offering predictable and easily obtainable food sources with lower foraging costs (Sıkdokur et al., 2024; Bautista et al., 2023; Kemahlı et al., 2023). Brown bears rely on forested habitats for cover, foraging, and denning (Recio et al., 2021; Ziółkowska et al., 2016); so they were included in the model. Population Density and Global Human Modification of Terrestrial Systems are important to understand the potential role of human dominancy in bear distribution (Sharma et al., 2022). Global Human Modification of Terrestrial Systems serves as an indicator of cumulative human pressure on the natural environment (Sharma et al., 2022). Topographic variables called Elevation, Slope, Aspect, and TRI were included in the model since they are highly associated with human land usage as well as bear habitat selection (Goldstein et al., 2010; Piédallu et al., 2019; Suel, 2019; Zarzo-Arias et al., 2019). Temperature and precipitation-related variables also drive the abundance, richness, and accessibility of natural prey and food sources of brown bears (Zarzo-Arias et al., 2021; Pérez-Girón et al., 2022; Pérez-Girón et al., 2024; Penteriani et al., 2019; Roberts et al., 2014; Garcia-Mozo et al., 2012; Afif-Khouri et al., 2011; Caprio & Quamme, 2011; Bautista et al., 2023).Furthermore, brown bears can change their food preferences depending on temperature fluctuations (Zarzo‐Arias et al., 2021; Kozakai et al., 2011; Deacy et al., 2017). Besides, temperature can modulate factors influencing the length of hyperphagia or cub-rearing season and entry/exit times of denning (González-Bernardo et al., 2020), affecting the likelihood of using particularly human dominated areas. Similarly, the precipitation-related predictors affect both bear foraging strategy and human activities (Garcia-Mozo et al., 2012; Afif-Khouri et al., 2011;Su et al., 2018; Pigeon et al., 2016; Dagtekin et al., 2024; Parsons et al., 2023).

**S.2 Future forecasting**

For future projections, bioclimatic variables with a resolution of 2.5 arc-minutes were downloaded from WorldClim 2.1, based on the CMIP6 (Coupled Model Intercomparison Project Phase 6) data, as featured in the sixth assessment report (AR6) of the Intergovernmental Panel on Climate Change. Three widely used general circulation models (GCMs); MIROC6, CNRM-CM6- 1, and MPI-ESM1-2-HR with three Shared Socio-economic Pathways/Representative Concentration Pathway scenarios including SSP1/RCP2.6 as optimistic future, SSP3/RCP7.0 as intermediate future and SSP5/RCP8.5 as pessimistic future were downloaded for both 2050 (average for 2041-2060) and 2070 (average for 2061-2080). These GCMs were chosen since they were already experienced in predicting accurate distribution modeling across Europe in other studies (Cantürk & Kulaç, 2021; Gür, 2022; Naimi et al., 2022).

**S.3 Habitat suitability modeling**

To predict both current and future distributions of brown bears across Türkiye, we employed an ensemble forecasting approach using the *biomod2* package (Thuiller et al., 2009). *Biomod2* is a versatile modeling platform capable of distinct modeling algorithms, resulting in an ensemble model. We applied six statistical and machine learning models, including the Generalized Linear Model (GLM), Maxent, Artificial Neural Network (ANN), Random Forest (RF), Generalized Boosted Model (GBM), and Extreme Gradient Boosting Training (XGBoost). GLMs extend simple linear regression by allowing the response variable to be modeled as a weighted linear function of multiple predictors (McCullagh, 2019). They can handle binary data, such as presence/(pseudo)absence, by maximizing likelihood to estimate weights that best explain species occurrences. Maxent is a machine learning algorithm that uses presence data and background locations to predict species distributions (Phillips et al., 2017). It identifies environmental profiles that best separate presence locations from the background, considering the environmental constraints of known occurrences. Inspired by biological neural systems, ANN consists of interconnected nodes arranged in layers: an input layer for data, one or more hidden layers for processing, and an output layer for predictions. ANN excels at capturing complex, nonlinear relationships in data (Lek & Guégan, 1999). RF is a tree-based ensemble method that uses bagging (bootstrap aggregation) to combine predictions from multiple decision trees (Breiman, 2001). Unlike boosting methods, RF randomly selects subsets of predictors at each split, making it less sensitive to hyperparameter tuning and a robust option for SDM. GBM builds an ensemble of decision trees sequentially, where each tree focuses on correcting the residual errors of the previous ones (De'Ath, 2007). This iterative approach improves predictions by targeting poorly modeled observations. Lastly, XGBoost is an advanced gradient boosting algorithm designed for scalability and regularization, which helps mitigate overfitting (Chen, 2015). Although its application in SDM is relatively recent, it shows promise due to its efficiency and flexibility in handling complex datasets. In summary, ANN and GLM utilize both presence and (pseudo)absence data, handling continuous and categorical predictors effectively. Tree-based models (RF, GBM, XGBoost) and MaxEnt, while differing in methodology, are particularly suited for ecological modeling due to their ability to capture nonlinearities and interactions in environmental data (Sillero et al., 2021).

The modeling process began with the random generation of 10,000 pseudo-absence points (Barbet-Massin et al., 2012). Subsequently, the dataset was split into training (70%) and testing (30%) datasets (Pant et al., 2021). We conducted three cross-validation iterations using a bootstrap approach for each model, and pseudo-absence points were regenerated ten times to mitigate random bias.

To assess model performance, we employed the true skill statistic (TSS), a prevalence-independent metric for species data (Allouche et al., 2006). TSS considers both omission and commission errors, yielding values between -1 (no better than random) and +1 (perfect agreement), with an AUC (Area Under the Curve) value exceeding 0.9 indicating excellent model performance. We selected models with TSS scores greater than 0.6 to reduce uncertainty and generate a final model, using a weighted average approach (Araújo and New, 2007; Thuiller et al., 2009).

Subsequently, we produced nineteen ensemble maps encompassing three distinct GCMs and scenarios for two time periods (2050s and 2070s) in addition to the present day. Binary transformation was performed using a threshold that maximizes TSS to create predictive outputs (Thuiller et al., 2019). To analyze changes in the range size between current and future scenarios, we generated output rasters depicting gain (range expansion), stable (no change), no occupancy, and loss (range contraction) areas through a comparative analysis of binary maps. To quantify changes in habitat range by latitude, we utilized the mean of the three future binary rasters for each scenarios and years (the cells have ≥0.5 value are counted as suitable).

We randomly generated 1,000 points for suitable areas across scenarios, and we considered mean values of these points for longitude and latitude to assess the spatial variance of suitable habitats. Additionally, we categorized averaged suitable habitats across scenarios into four elevation ranges (<1,000 m; 1,000-2,000 m; 2,000-3,000 m; >3,000m) to investigate altitude-related shifts in suitable patches. We examined the land-cover proportions of suitable areas for present-day as well as across scenarios by using averaged rasters of three GCMs.

Finally, the status of established protected areas was evaluated for both current and future projections, based on the above-mentioned rasters. There are various protected area attributes in Türkiye (Atmiş, 2018; NCNP, 2022); however, the related protected area attributes in terms of brown bear ecology were selected and extracted as follows: *national parks, protection forests, city forests, gene conservation forests, species environment protected areas, nature reserve areas, nature parks, national monuments, wildlife enhancement areas, Ramsar areas, nationally important wetlands,* and *locally important wetlands.*

**Table S1:** Environmental predictors used for ecological niche modeling of brown bears in türkiye. The bold variables were selected to run the final model.

| **Category** | **Predictors** | **Unit** | **Source** |
| --- | --- | --- | --- |
| Bioclimatic | **Annual mean temperature (Bio1)** | ^o^C | Worldclim |
|  | **Mean diurnal range (Bio2)** | ^o^C |  |
|  | Isothermality (Bio3) | Dimensionless (%)* |  |
|  | **Temperature seasonality (Bio4)** | ^o^C |  |
|  | Maximum temperature of warmest month (Bio5) | ^o^C |  |
|  | Minimum temperature of coldest month (Bio6) | ^o^C |  |
|  | Temperature annual range (Bio7) | ^o^C |  |
|  | **Mean temperature of wettest quarter (Bio8)** | ^o^C |  |
|  | **Mean temperature of driest quarter (Bio9)** | ^o^C |  |
|  | Mean temperature of warmest quarter (Bio10) | ^o^C |  |
|  | Mean temperature of coldest quarter (Bio11) | ^o^C |  |
|  | Annual precipitation (Bio12) | mm |  |
|  | **Precipitation of wettest month (Bio13)** | mm |  |
|  | **Precipitation of driest month (Bio14)** | mm |  |
|  | Precipitation seasonality (Bio15) | Dimensionless (%)* |  |
|  | Precipitation of wettest quarter (Bio16) | mm |  |
|  | Precipitation of driest quarter (Bio17) | mm |  |
|  | Precipitation of warmest quarter (Bio18) | mm |  |
|  | Precipitation of coldest quarter (Bio19) | mm |  |
| Topographic | Elevation | M | SRTM |
|  | **Slope** | degrees |  |
|  | **Aspect** | degrees |  |
|  | **Terrain ruggedness index (TRI)** | - |  |
| Anthropogenic | **Distance to forest** | m | MODIS/Terra+Aqua Land Cover |
|  | **Distance to cropland** | m |  |
|  | Distance to grassland | m |  |
|  | **Distance to build-up areas** | m |  |
|  | **Distance to water** | m | HydroRIVERS |
|  | **Distance to road** | m | ArcGIS World Roads |
|  | **Population density** | no. of persons/km^2^ | Global Human Settlement Layer |
|  | **Global human modification of terrestrial systems** | - | NASA EarthData |

*Isothermality (Bio3) and Precipitation Seasonality (Bio15) are dimensionless ratios. They are often expressed as percentages for interpretation but do not have physical units.

**Table S2:** Projected range change of brown bears across the country and biogeographic regions for three GCMs and their average in 2050 and 2070 under different scenarios.

| **GCMs** | **Scenario** | **Loss (%)** | **Gain (%)** | **Range Change** | **Loss (%)** | **Gain (%)** | **Range Change** | **Loss (%)** | **Gain (%)** | **Range Change** | **Loss (%)** | **Gain (%)** | **Range Change** |
| --- | --- | --- | --- | --- | --- | --- | --- | --- | --- | --- | --- | --- | --- |
| **2050** |  | **Euro-Siberian** | | | **Irano-Turanian** | | | **Mediterranean** | | | **Whole Country** | | |
| **MIROC6** | **RCP2.6** | 54 | 4 | -49.8 | 66 | 3 | -63 | 61 | 1 | -59.2 | 60 | 3 | -56.5 |
|  | **RCP7.0** | 52 | 4 | -47.9 | 59 | 3 | -55.9 | 59 | 2 | -57.2 | 56 | 4 | -52.4 |
|  | **RCP8.5** | 59 | 4 | -54.8 | 75 | 2 | -72.8 | 69 | 1 | -67.8 | 67 | 3 | -64 |
| **CNRM-CM6-1** | **RCP2.6** | 42 | 10 | -32.1 | 51 | 7 | -43.8 | 55 | 2 | -52.4 | 47 | 8 | -39.4 |
|  | **RCP7.0** | 47 | 8 | -38.5 | 53 | 10 | -43.8 | 59 | 3 | -56.1 | 51 | 8 | -42.8 |
|  | **RCP8.5** | 52 | 8 | -44.1 | 60 | 10 | -50 | 63 | 2 | -60.8 | 57 | 8 | -48.5 |
| **MPI-ESM1-2-HR** | **RCP2.6** | 20 | 17 | -3.1 | 19 | 22 | 3 | 30 | 8 | -22 | 21 | 18 | -2.6 |
|  | **RCP7.0** | 29 | 18 | -11.2 | 33 | 19 | -14.5 | 43 | 5 | -38.3 | 32 | 17 | -15.7 |
|  | **RCP8.5** | 29 | 18 | -10.8 | 36 | 18 | -17.2 | 46 | 5 | -41.5 | 34 | 17 | -17 |
| **Average** | **RCP2.6** | **42** | **9** | **-33** | **51** | **7** | **-43.9** | **53** | **2** | **-51.1** | **47** | **7** | **-39.8** |
|  | **RCP7.0** | **45** | **8** | **-37.6** | **50** | **8** | **-41.4** | **56** | **2** | **-53.4** | **48** | **7** | **-41** |
|  | **RCP8.5** | **50** | **8** | **-42.5** | **59** | **8** | **-50.6** | **61** | **2** | **-58.9** | **55** | **7** | **-47.9** |
| **2070** | **Scenario** | **Loss (%)** | **Gain (%)** | **Range Change** | **Loss (%)** | **Gain (%)** | **Range Change** | **Loss (%)** | **Gain (%)** | **Range Change** | **Loss (%)** | **Gain (%)** | **Range Change** |
| **MIROC6** | **RCP2.6** | 54 | 5 | -49.1 | 68 | 2 | -65.9 | 63 | 1 | -61.8 | 61 | 3 | -57.8 |
|  | **RCP7.0** | 57 | 7 | -50.6 | 73 | 3 | -70.7 | 74 | 2 | -72.5 | 66 | 4 | -61.7 |
|  | **RCP8.5** | 74 | 2 | -71.9 | 91 | 1 | -90.6 | 86 | 2 | -84.1 | 83 | 2 | -81.3 |
| **CNRM-CM6-1** | **RCP2.6** | 38 | 11 | -26.6 | 50 | 10 | -39.5 | 55 | 3 | -52.1 | 45 | 10 | -35.1 |
|  | **RCP7.0** | 52 | 10 | -42.3 | 67 | 12 | -54.9 | 68 | 4 | -64.4 | 60 | 10 | -50.2 |
|  | **RCP8.5** | 65 | 6 | -59.5 | 81 | 7 | -73.5 | 80 | 4 | -76.3 | 74 | 6 | -67.4 |
| **MPI-ESM1-2-HR** | **RCP2.6** | 35 | 8 | -26.7 | 31 | 10 | -21.5 | 39 | 2 | -36.8 | 34 | 8 | -25.6 |
|  | **RCP7.0** | 41 | 16 | -24.7 | 46 | 19 | -26.3 | 55 | 5 | -49.8 | 45 | 16 | -28.2 |
|  | **RCP8.5** | 44 | 16 | -28.4 | 54 | 17 | -37.2 | 69 | 3 | -65.5 | 51 | 15 | -36.4 |
| **Average** | **RCP2.6** | **42** | **7** | **-34.4** | **49** | **6** | **-42.7** | **55** | **2** | **-53.1** | **46** | **6** | **-40.1** |
|  | **RCP7.0** | **50** | **10** | **-39.3** | **64** | **10** | **-54** | **67** | **3** | **-64.2** | **58** | **9** | **-48.5** |
|  | **RCP8.5** | **63** | **6** | **-57.2** | **81** | **6** | **-74.2** | **80** | **3** | **-76.8** | **72** | **6** | **-66.7** |

**Table S3: Change in the mean elevation in suitable habitats for brown bears shortly under various climate change scenarios (average of three GCMs).**

| **Time** | **Scenario** | Mean Altitde (m) | Difference (m) |
| --- | --- | --- | --- |
| **Present day** | **-** | 1930.99 | - |
| **2050** | **RCP2.6** | 2098.13 | 167.14 |
|  | **RCP7.0** | 2171.26 | 240.27 |
|  | **RCP8.5** | 2176.46 | 245.47 |
| **2070** | **RCP2.6** | 2108.12 | 177.13 |
|  | **RCP7.0** | 2237.67 | 306.68 |
|  | **RCP8.5** | 2266.04 | 335.04 |


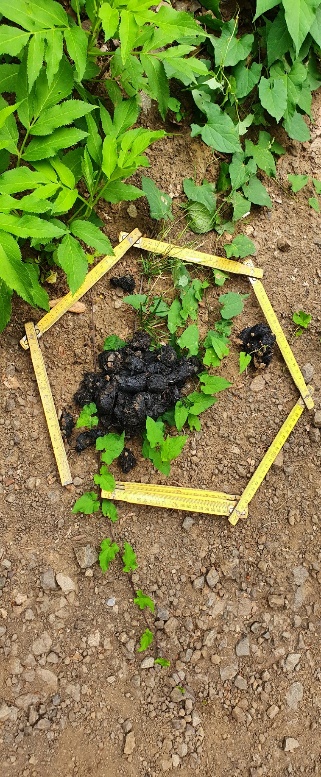

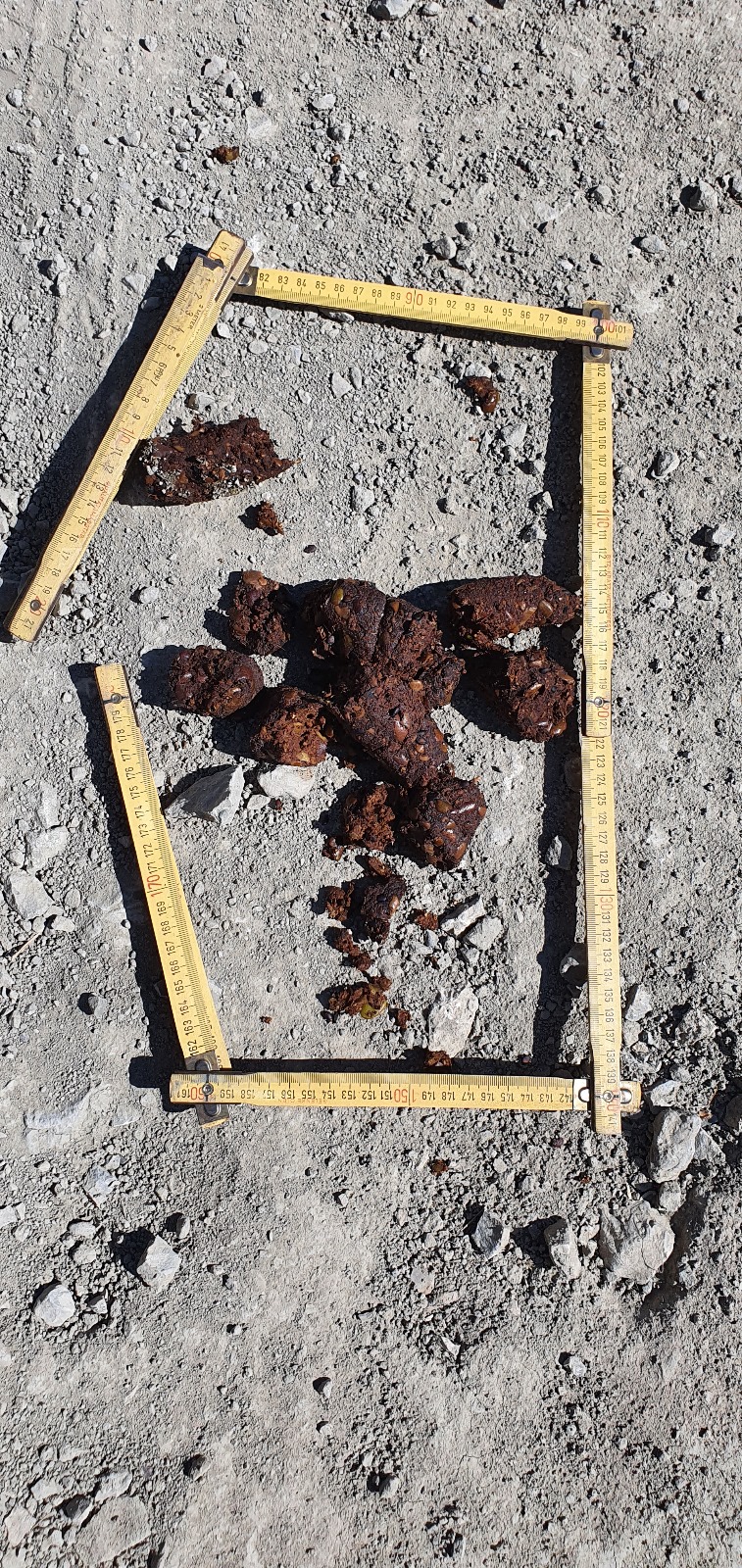

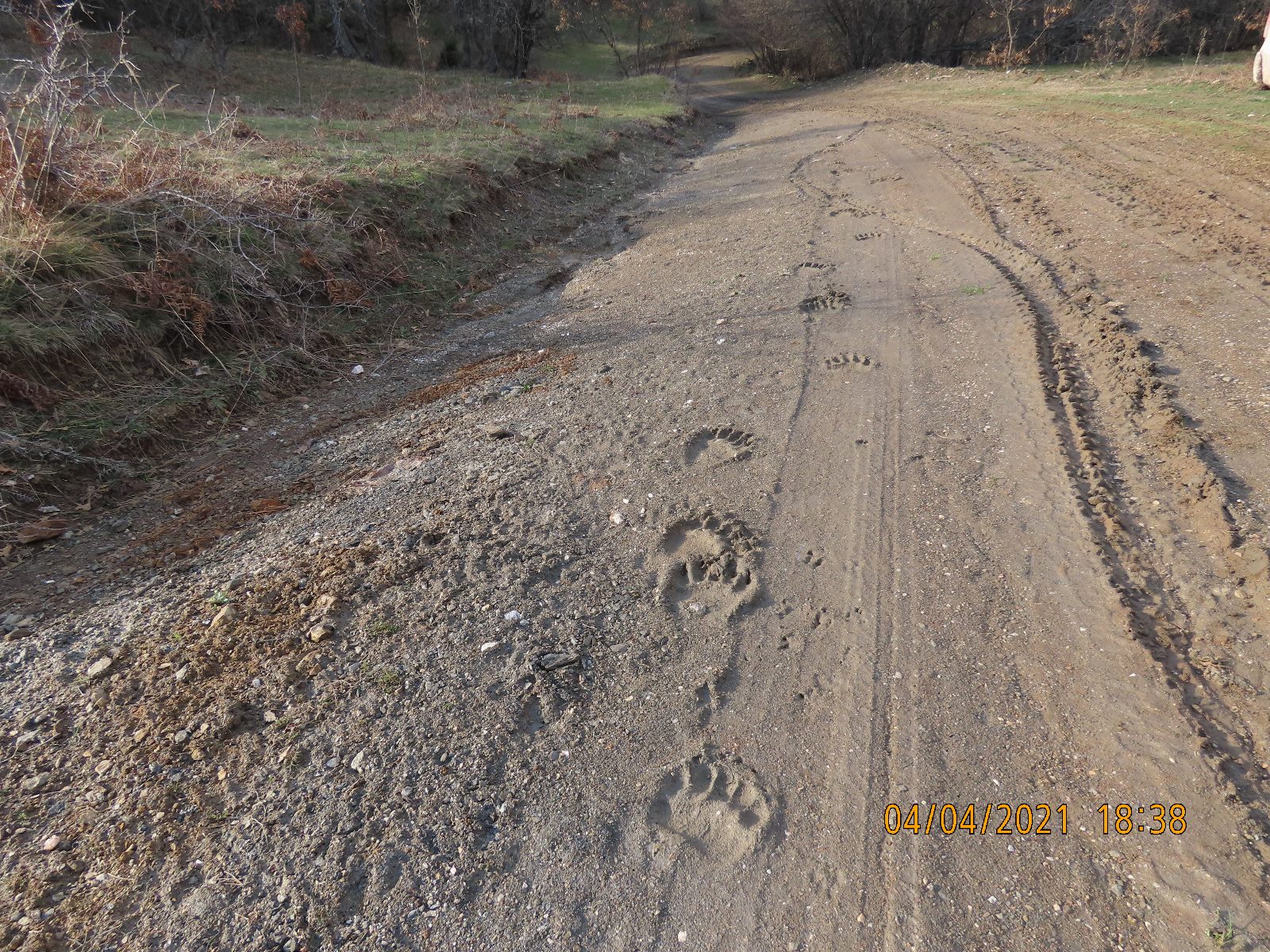

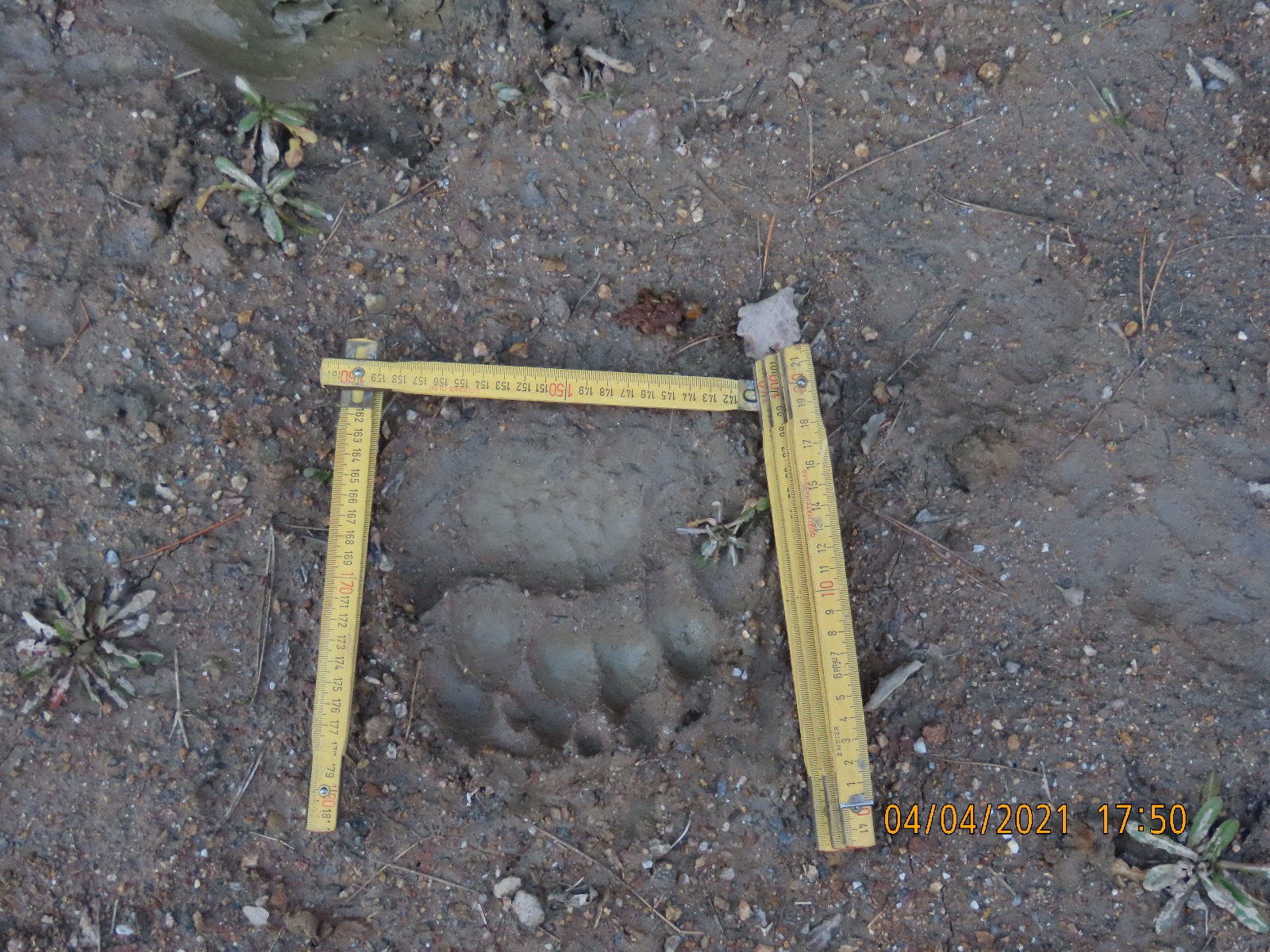

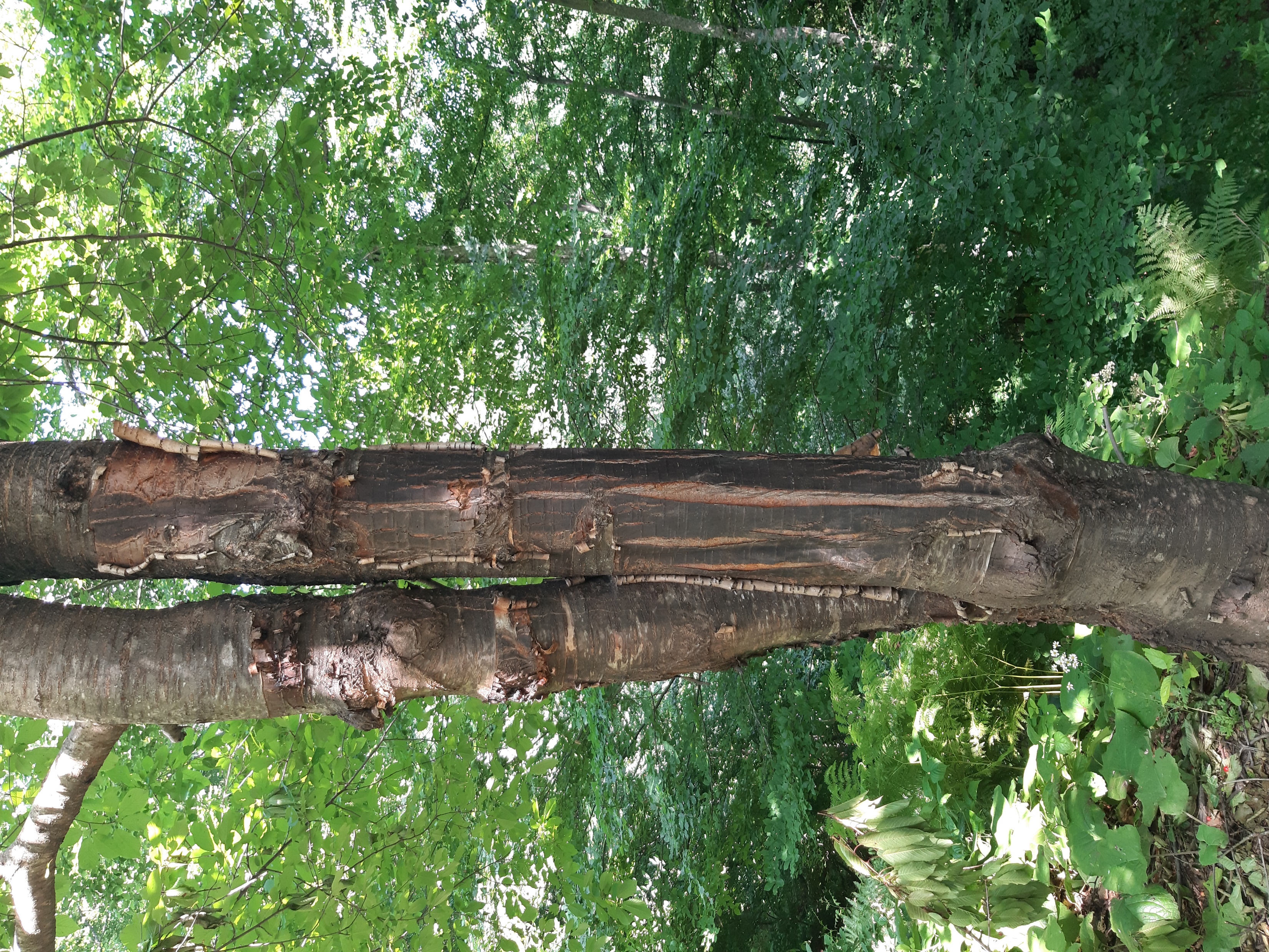

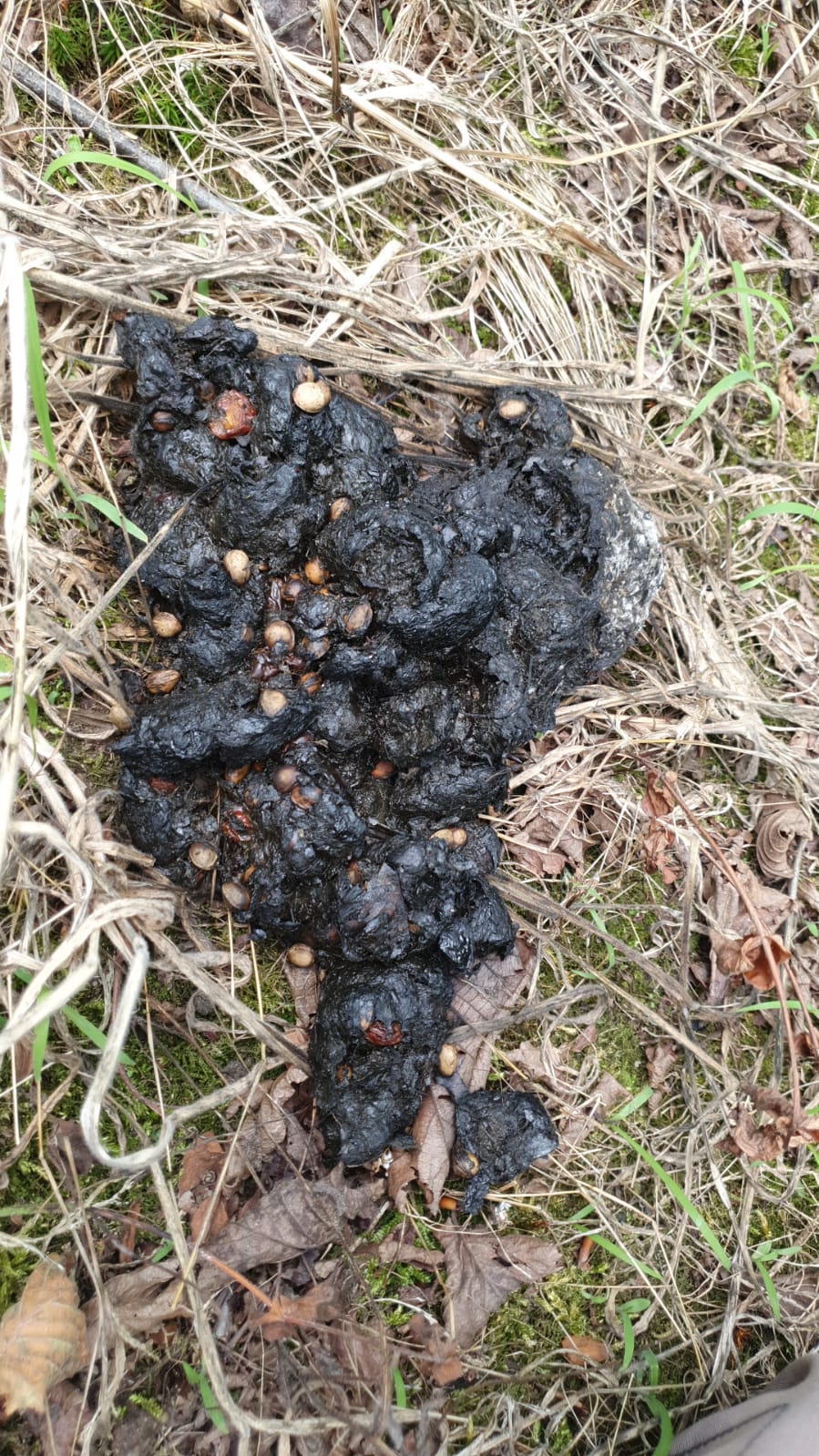


**Fig. S1:** Presence signs of brown bears including a rubbing tree, footprints, and scat samples.


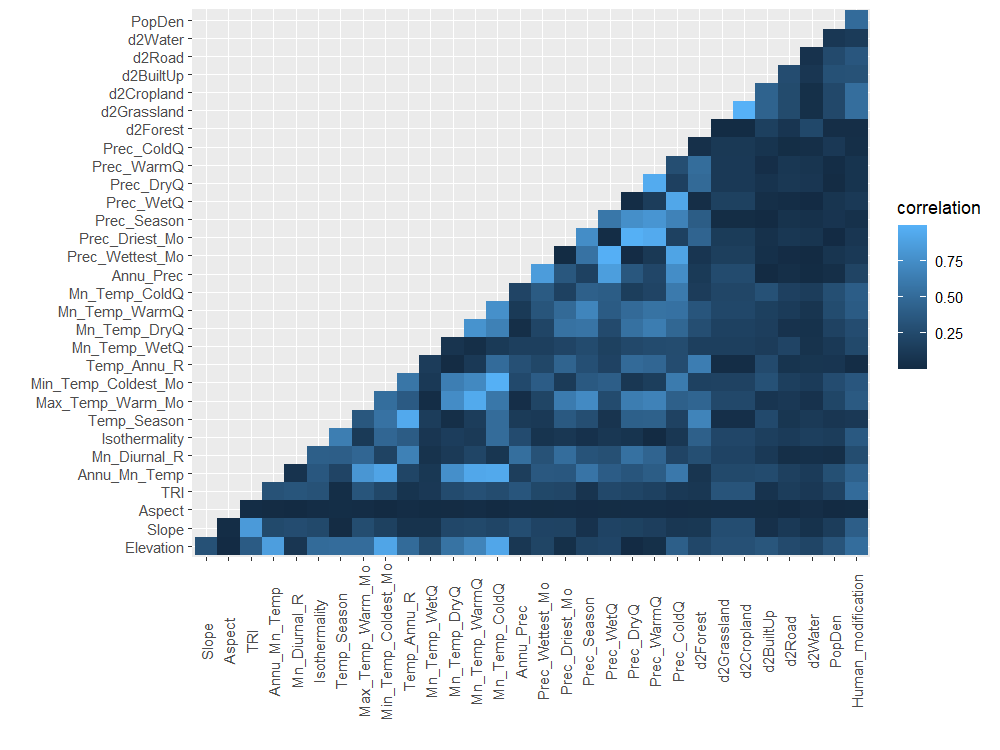


**Fig. S2:** Correlation matrix of environmental predictors for ecological niche modeling.


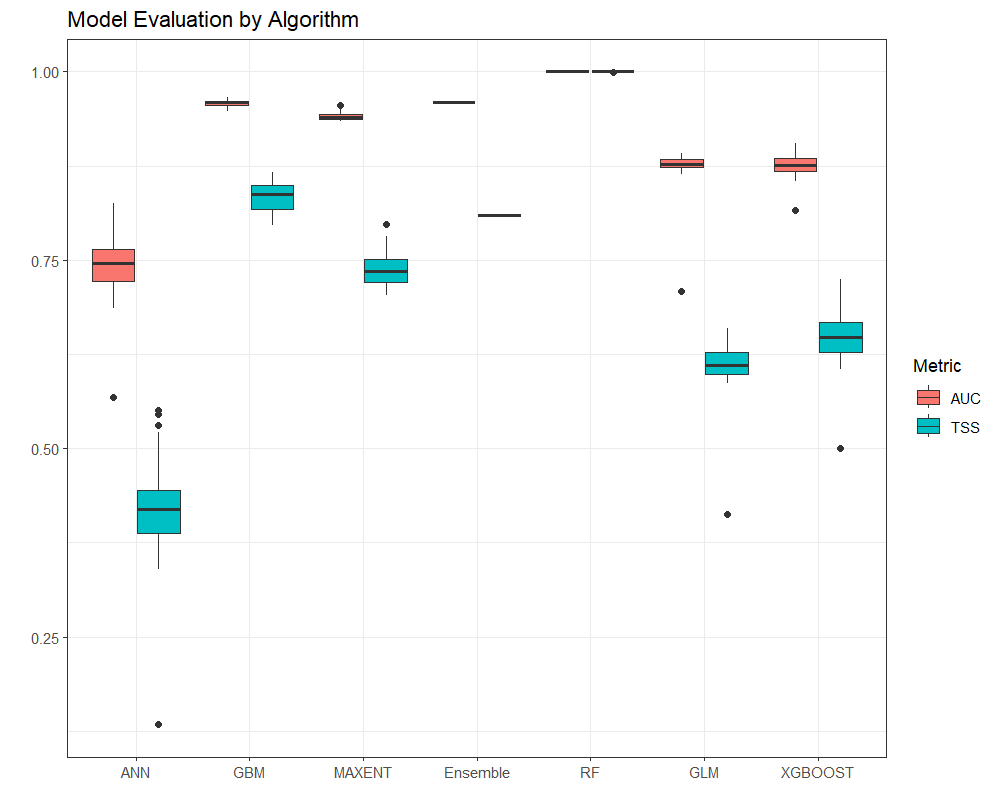


**Fig. S3:** Receiver operating characteristic (ROC) area under the curve (AUC) and True skill statistics (TSS) values as the predictive performance of the different modeling techniques used in the ecological niche modeling.

The six ecological niche models evaluated in this study showed varying levels of predictive accuracy. Among them, RF, GBM, and XGBoost emerged as the most effective, with the highest AUC and TSS values. The ensemble model, which integrated the top-performing algorithms, demonstrated outstanding predictive capability, achieving an AUC of 0.96 and a TSS of 0.81.


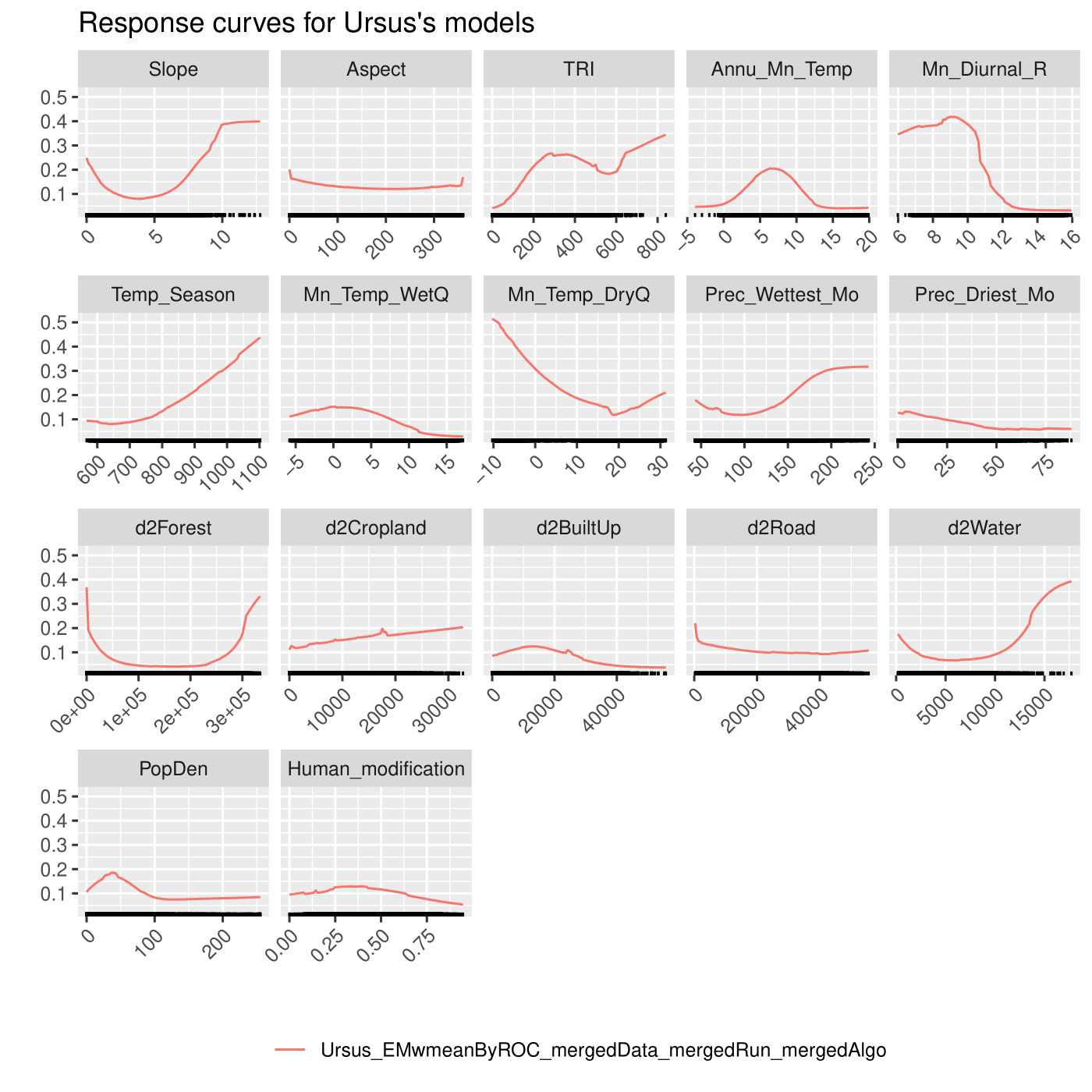


**Fig. S4:** Response curves of the environmental predictors in the ensemble model for ecological niche modeling of brown bears across Türkiye.


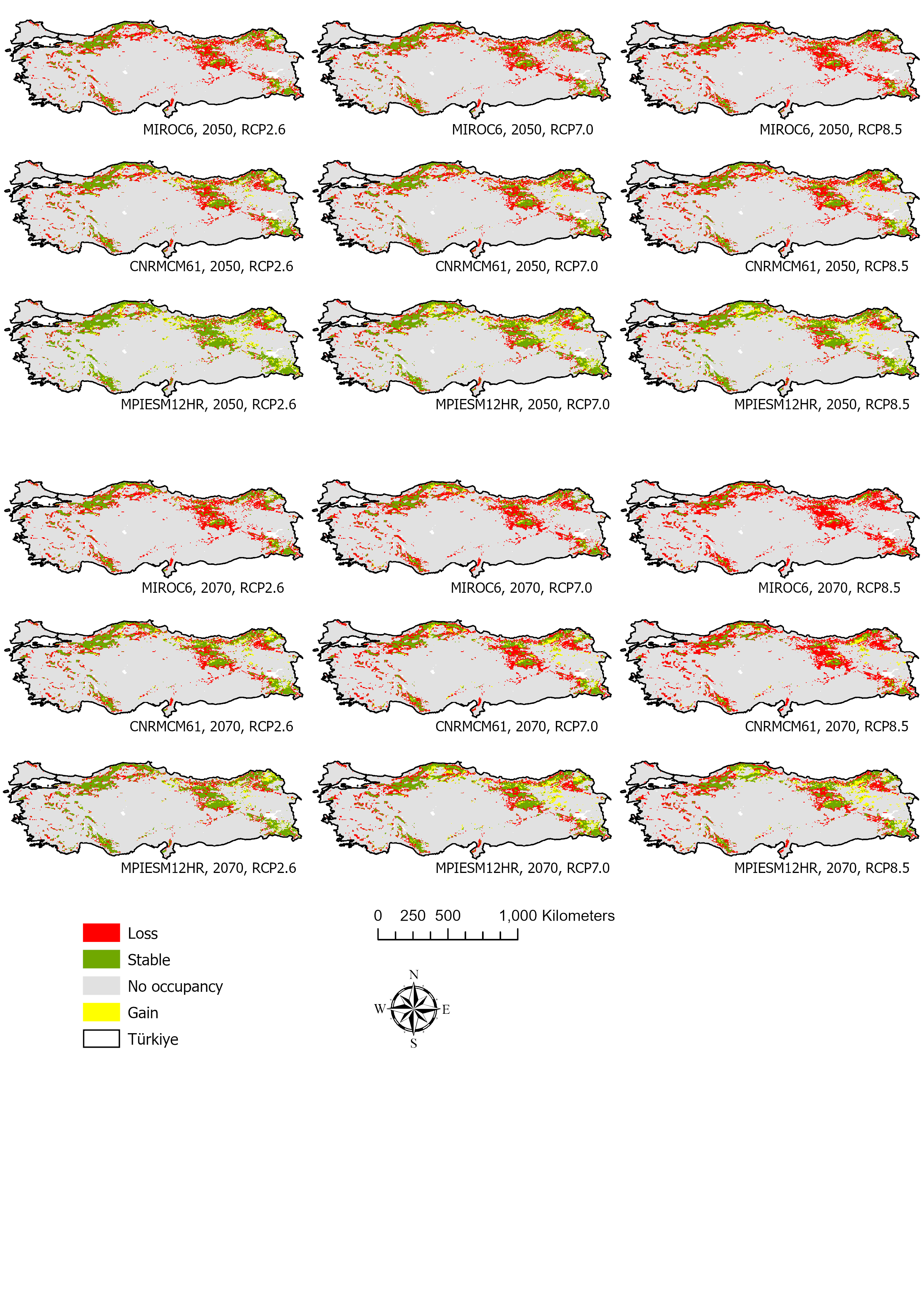


**Fig. S5:** Ensemble forecasting of the future range change by considering optimistic (RCP 2.6), intermediate (RCP 7.0), and pessimistic (RCP 8.5) scenarios for brown bears of Türkiye in both 2050 and 2070 based on MIROC6, CNRM-CM6-1, and MPI-ESM1-2-HR GCMs.


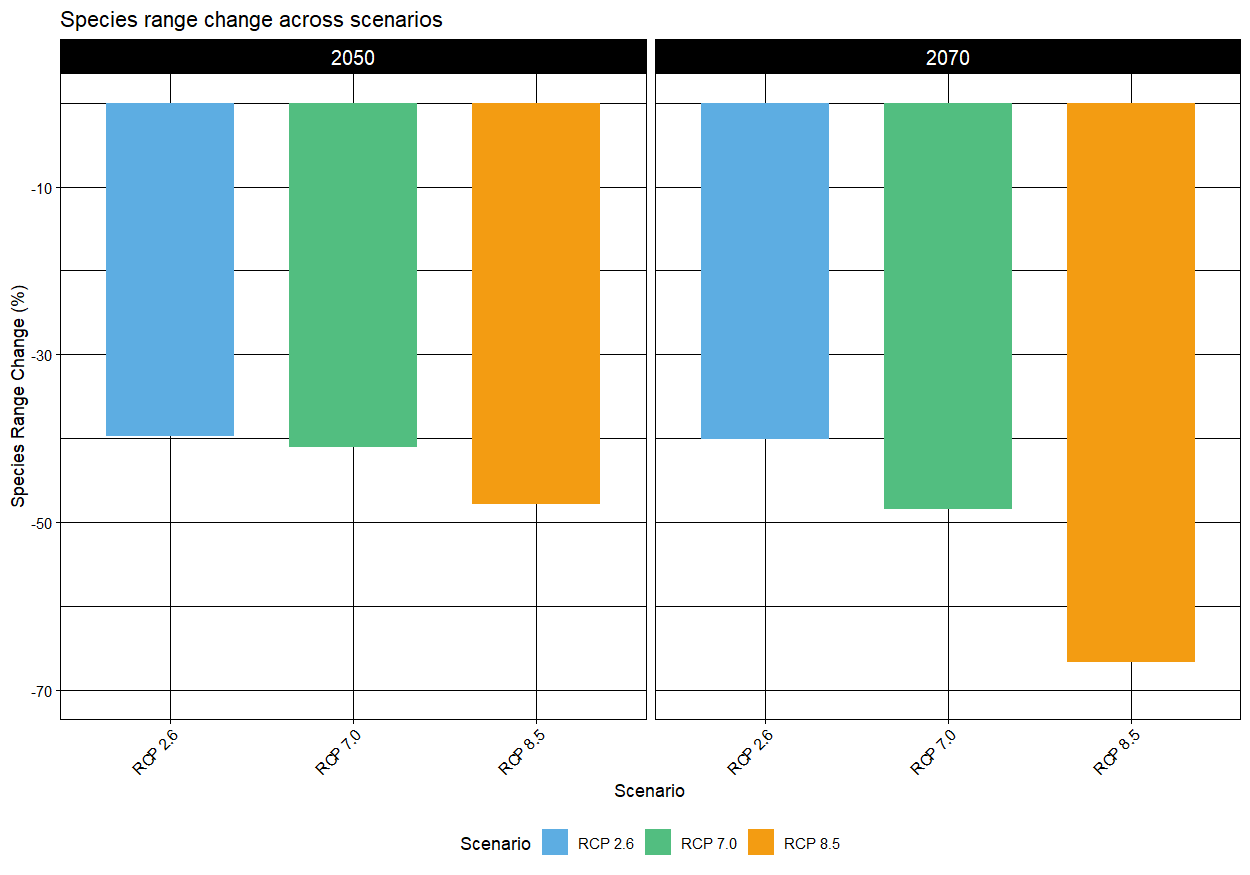


**Fig. S6:** Brown bears’ range change in Türkiye across scenarios and years, based on the average rasters of three GCMs (MIROC6, CNRM-CM6-1, MPI-ESM1-2-HR).


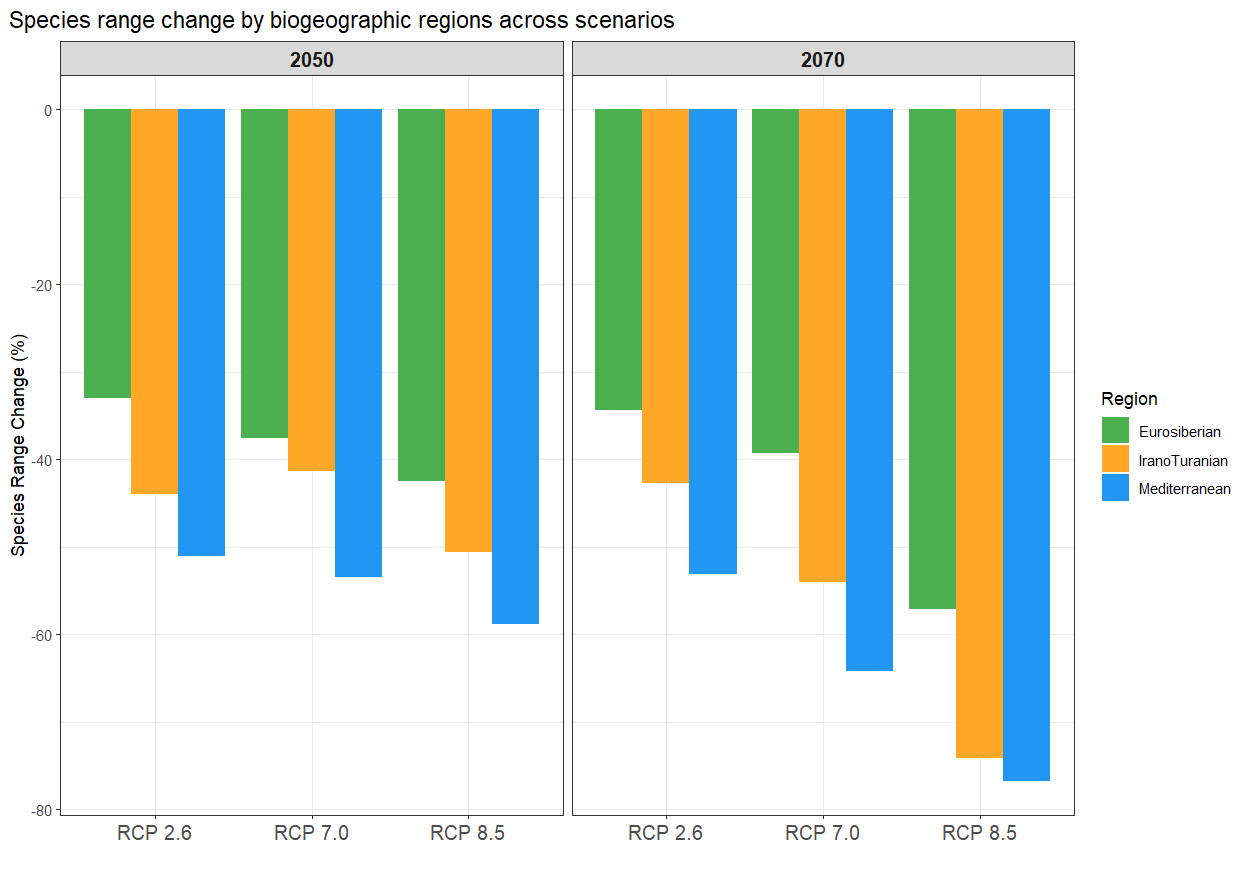
**Fig. S7:** Brown bears’ range change in different biogeographic regions of Türkiye across scenarios and years, based on the average rasters of three GCMs (MIROC6, CNRM-CM6-1, MPI-ESM1-2-HR).


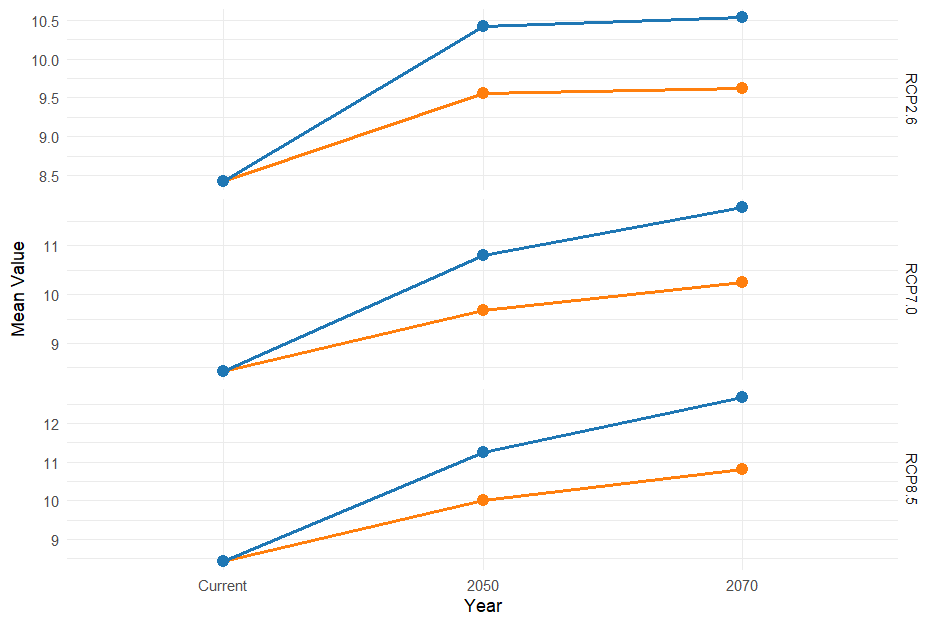


**Fig. S8:** Projected annual mean temperature values for brown bears’ suitable habitats in the near future. The blue line represents the annual mean temperature values faced by brown bears if they continue to occupy their current suitable patches, while the orange line depicts the values for future suitable patches. For each climate change scenario, the average of three GCMs was considered.

**The checklist for ODMAP (Overview, Data, Model, Assessment, and Prediction) protocol for ecological niche modeling of Eurasian brown bears (Ursus arctos) across Türkiye**

| **ODMAP Elements** | **Contents** | |
| --- | --- | --- |
| **Overview** | *Model objective* | **Objective**: Ecological niche modeling (ENM) of brown bears across Türkiye.  **Target outputs**: Potential current and future habitat suitability maps. |
|  | *Taxon* | Brown bear, *Ursus arctos*, Ursidae, Carnivora, Mammalia |
|  | *Location* | Türkiye, Soutwest Asia |
|  | *Scale of analysis* | **Spatial extent**: 25.65708, 44.83208, 35.82458, 42.10792 (xmin, xmax, ymin, ymax)  **Temporal extent**:  Occurrence records – from 2020 to 2021.  Bioclimatic predictors (WorldClim) – from 1970 to 2000.  Forest, Cropland, Grassland, Build-up areas: from 2018.  Water layers – from 2018.  Roads – from 2023.  Population density – from 2020.  Global Human Modification of Terrestrial Systems – from 2016.  **Type of extent boundary**: Political (Republic of Türkiye) |
| **Data** | *Biodiversity data overview* | **Observation type:** Presence points including footprints, scat samples, hair/tissue samples, rubbing tree signs (n=608)  **Response/Data type:** Presence-only |
|  | *Type of predictors* | Bioclimatic, Topographic, Anthropogenic |
|  | *Conceptual model / hypothesis* | Brown bear distribution is driven by climatic, topographic and human-related factors. |
|  | *Data collection* | **Data source:** Between May 2020 and October 2021, extensive fieldwork was conducted to gather data on brown bear presence signs across Türkiye.  **Absence-data:** Presence-only data were used for modeling. Pseudo-absence generation was performed using *biomod2* (Thuiller et al., 2009).  **Data filtering:** *spThin* package (Aiello-Lammens et al., 2015) were used to avoid spatial clustering of occurrence records. |
|  | *Predictor variables* | **Variables**  **Bioclimatic Variables**:  Bioclimatic variables – BIO1: Annual mean temperature (^o^C), BIO2: Mean diurnal range (^o^C), BIO3: Isothermality, BIO4: Temperature seasonality (^o^C), BIO5: Max. Temperature of warmest month (^o^C), BIO6: Min. temperature of coldest month (^o^C), BIO7: Temperature annual range (^o^C), BIO8: Mean temperature of wettest quarter (^o^C), BIO9: Mean temperature of driest quarter (^o^C), BIO10: Mean temperature of warmest quarter (^o^C), BIO11: Mean temperature of coldest quarter (^o^C), BIO12: Annual precipitation (mm), BIO13: Precipitation of wettest month (mm), BIO14: Precipitation of driest month (mm), BIO15: Precipitation seasonality, BIO16: Precipitation of wettest quarter (mm), BIO17: Precipitation of driest quarter (mm), BIO18: Precipitation of warmest quarter (mm), BIO19: Precipitation of coldest quarter (mm)  **Tographic Variables:**  Elevation (m), Slope (degrees), Aspect (degrees), Terrain Ruggedness Index (TRI)  **Anthropogenic Variables:**  Distance to forest (m), Distance to cropland (m), Distance to grassland (m), Distance to build-up areas (m), Distance to water (m), Distance to road (m), Population density (no. of persons/km^2^), Global Human Modification of Terrestrial Systems  **Data Sources**  Worldclim (<https://www.worldclim.org/>)  MODIS/Terra+Aqua Land Cover Type Yearly L3 Global 500 m SIN Grid database (doi:10.5067/MODIS/MCD12Q1.006)  ArcGIS World Roads layer (<https://hub.arcgis.com/maps/Story::world-roads/about>)  HydroRIVERS – HydroSHEDS (<https://www.hydrosheds.org/products/hydrorivers>)  Global Human Settlement Layer (<https://human-settlement.emergency.copernicus.eu/download.php?ds=pop>)  Global Human Modification of Terrestrial Systems (<https://doi.org/10.7927/edbc-3z60>)  **Spatial resolution of raw data:**  Worldclim (2.5 arc-min), Global Human Settlement Layer (30 arc-sec), Global Human Modification of Terrestrial Systems (30 arc-sec), BIO1-19 (2.5 arc-min), MODIS/Terra+Aqua Land Cover Type Yearly L3 Global 500 m SIN Grid (1200 km x 1200 km), Elevation (2.5 arc-min), HydroRIVERS (15 arc-sec)  **Projection:** WGS84  **Data Processing:**  Bioclimatic variables were downloaded from WorldClim database with 2.5 arc-min resolution (approximately 4.6 km at the equator). These layers were masked and cropped according to Turkiye’s border (<https://gadm.org/download_country36.html>).  The forest layer was generated by using the MODIS/Terra+Aqua Land Cover Type Yearly L3 Global 500 m SIN Grid database. To do this, the attributes named *Evergreen Needleleaf Forests, Evergreen Broadleaf Forests, Deciduous Needleleaf Forests, Deciduous Broadleaf Forests, Mixed Forests* were combined and extracted. Then, the proximity layer called Distance to forest was generated using Euclidean Distance Tool in ArcGIS by arranging its cell-size and extent.  The cropland layer was generated by using the MODIS/Terra+Aqua Land Cover Type Yearly L3 Global 500 m SIN Grid database. To do this, the attributes named *Cropland*, and *Cropland/Natural Vegetation Mosaics* were combined and extracted. Then, the proximity layer called Distance to cropland was generated using Euclidean Distance Tool in ArcGIS by arranging its cell size and extent.  The Built-Up layer was generated by using the MODIS/Terra+Aqua Land Cover Type Yearly L3 Global 500 m SIN Grid database. To do this, the attributes named *Urban and Built-up Lands* extracted. Then, the proximity layer called Distance to Built-Up areas was generated using Euclidean Distance Tool in ArcGIS by arranging its cell size and extent.  The Grassland layer was generated by using the MODIS/Terra+Aqua Land Cover Type Yearly L3 Global 500 m SIN Grid database. To do this, the attributes named *Graslands* extracted. Then, the proximity layer called Distance to Grassland was generated using Euclidean Distance Tool in ArcGIS by arranging its cell size and extent.  To consder water resources, *1^st^ to 4^th^ order river* attributes were extracted from HydroRIVERS database since this vector layer appropriately covers important water sources across the country, from small springs in/around forests and agricultural fields to large rivers. Then, the proximity layer named Distance to water was generated by using the Euclidean Distance Tool in ArcGIS.  The attributes named *major roads* and *highways* were extracted from the ArcGIS World Roads Layer. Then, the proximity layer named Distance to roads was generated by using the Euclidean Distance Tool in ArcGIS.  Population Density data was downloaded from Global Human Settlement Layer database. The raster resampled, cropped, and masked based on the study area.  Global Human Modification of Terrestrial Systems data were downloaded from NASA EarthData website. The raster resampled, cropped, and masked based on the study area.  The Slope, Aspect and Terrain ruggedness index rasters were derived from the Shuttle Radar Topography Mission (SRTM) Elevation data. They were derived after projecting to Lambert Azimuthal Equal-Area (ETRS 1989 LAEA), and then projected WGS84 again (Sillero and Barbosa, 2021).  All variables were properly resampled, cropped, and masked based on the study area. |
|  | *Transfer data for projection* | All topographic and anthropogenic variables were kept static.  The bioclimatic variables with a resolution of 2.5 arc-minutes from WorldClim 2.1., based on the CMIP6 (Coupled Model Intercomparison Project Phase 6) data, as featured in the sixth assessment report (AR6) of the Intergovernmental Panel on Climate Change.  Three widely used GCMs; **MIROC6**, **CNRM-CM6-1**, and **MPI-ESM1-2-HR** since they were already used to predict accurate ENMs across Europe in other studies (Canturk & Kulaç, 2021; Gür, 2022; Naimi et al., 2022).  For all three GCMs, three different Shared Socio-economic Pathways/Representative Concentration Pathway scenarios including **SSP1/RCP2.6** as an optimistic future, **SSP3/RCP7.0** as a intermediate future and **SSP5/RCP8.5** as a pessimistic future were downloaded for both 2050 (average for 2041-2060) and 2070 (average for 2061-2080) to project future habitat suitability. |
| **Model** | *Assumptions* | We assumed that species are at equilibrium with the environment.  Occurrence records were collected by avoiding any observational bias during the fieldwork.  The filtering process is performed to avoid spatial clustering of occurrence records.  Predictor variables are incorporated in the model after checking any error.  Multicollinearity among the environmental predictors was checked. |
|  | *Algorithms* | Six SDM algorithms available in *biomod2* package were used.  Generalised Linear Model (GLM)  Artificial Neural Network (ANN)  Maxent Entropy (Maxent)  Random Forest (RF)  Generalised Boosting Model (GBM)  Extreme Gradient Boosting Training (XGBOOST)  **Model complexity:** These algorithms were chosen to yield complex response surfaces while preventing overfitting. |
|  | *Model workflow* | *spThin* package (Aiello-Lammens et al., 2015) were used to filter the spatial clustering of points based on 10 km across the study area. 121 presence records were used as the final dataset for modeling.  Thirty-one environmental variables, including bioclimatic, topographic, and anthropogenic predictors that have a high potential to drive brown bear distribution (Su et al., 2018; Dai et al., 2019; Dai et al., 2021; Dar et al., 2021; Mukherjee et al., 2021, Mohammadi et al., 2021; Ashrafzadeh et al., 2022).  Multicollinearity among variables were checked and excluded the variables with correlation coefficients (\|r\|) >0.7 and variance inflation factor (VIF) > 5 by using *usdm* and *corrplot* package in the R environment (Wei et al., 2021; Naimi, 2023). Ultimately, 17 environmental variables were used to perform modeling.  To predict both potential current distribution and future habitat suitability of brown bears across Türkiye, an ensemble forecasting approach were employed using the *biomod2* package.  The modeling process began with the random generation of 10,000 pseudo-absence points.  Subsequently, the dataset was split into training (70%) and testing (30%) datasets.  Three cross-validation iterations were conducted using a bootstrap approach for each model.  Pseudo-absence points were regenerated ten times to mitigate random bias.  To reduce uncertainty and generate a final model, the models with TSS scores greater than 0.6 were selected and used weighted average approach (Araújo and New, 2007; Thuiller et al., 2009).  Future forecasting were performed with the ensemble model by considering three different CO_2_ scenarios for two different future time, 2050 and 2070.  Nineteen ensemble maps were produced encompassing three distinct GCMs and scenarios for two time periods (2050s and 2070s) in addition to the present day.  Binary transformation was performed using a threshold that maximizes TSS to create predictive outputs (Thuiller et al., 2019).  To analyze changes in the range size between current and future scenarios, the range size function within the *biomod2* package was employed. |
|  | *Software, codes, and data* | **Modeling platform:** R (*version 4.3.0*) environment with *biomod2* package.  **Dataset and Code:** The datasets used and/or the R codes during the current study are available on reasonable request. |
|  | *Data partioning* | Occurrence records were split out as training (70%) and testing (30%) datasets. |
|  | *Variable selection* | All environmental variables that have a high potential to drive brown bear distribution were selected based on the literature. For instance, roads, human settlements, and croplands can represent important ecological traps and affect bears’ foraging behavior as well as population persistence (Lamb et al., 2017; Parsons et al., 2023).  Brown bears rely on forested habitats for cover, foraging, and denning (Recio et al., 2021; Ziółkowska et al., 2016); so they were included in the model. Population density and Global Human Modification of Terrestrial Systems are important to understand the potential role of human dominancy in their distribution (Dai et al., 2019). Topographic variables called Elevation, Slope, Aspect, and TRI were included in the model since they are highly associated with human land usage as well as bear ecology (Goldstein et al., 2010). Bioclimatic variables also drive the abundance, richness, and accessibility of natural prey and food sources of brown bears (Zarzo-Arias et al., 2021; Pérez-Girón et al., 2022; Pérez-Girón et al., 2024; Penteriani et al., 2019).  Multicollinearity were checked using *usdm* and *corrplot* packages among the variables and excluded them if they displayed high correlation (correlation coefficient > 0.7) and variance inflation (VIF >5). Ultimately, 17 variables were retained. |
|  | *Model settings* | Reproducing ENM results is important and depends on the processing of occurrence data, environmental data and their processing, model calibration, and repeatability of model transfer processes (Feng et al., 2019). Therefore, the default settings of the algorithms were selected to simplify the interpretation and reproducibility of the results. |
| **Assessment** | *Model estimates* | True Skill Statistics was employed to assess the discrimination capacity of the model. ROC was taken into account for the occupy vs. non-occupy comparison.  The variable importance of the ensemble model was calculated. |
|  | *Plausibility check* | The ensemble model's response curve and the predictors' ecological plausibility were checked. |
|  | *Ensemble model* | The models with TSS scores greater than 0.6 were selected using a weighted average approach to generate an ensemble model (Araújo and New, 2007). |
|  | *Non-independence* | None |
|  | *Threshold* | TSS maximization threshold was considered for binary predictions. |
|  | *Performance statistics* | Model performance were evaluated based on TSS value from 246 models. |
| **Prediction** | *Prediction output* | Continuous predictions of occurrence probability for brown bears living in Türkiye were used. |
|  | *Uncertainty quantification* | The ensemble approach significantly increases the prediction performance by combining results from different modeling techniques and thus presents the relationships that are aimed to be captured by the model by separating them from the background noise (Hao et al., 2020). |

**References**

Afif-Khouri, E., Álvarez-Álvarez, P., Fernández-López, M. J., Oliveira-Prendes, J. A., & Cámara-Obregón, A. (2011). Influence of climate, edaphic factors and tree nutrition on site index of chestnut coppice stands in north-west Spain. *Forestry*. https://doi.org/10.1093/forestry/cpr025

Aiello-Lammens, M. E., Boria, R. A., Radosavljevic, A., Vilela, B., & Anderson, R. P. (2015). spThin: An R package for spatial thinning of species occurrence records for use in ecological niche models. *Ecography*. https://doi.org/10.1111/ecog.01132

Allouche, O., Tsoar, A., & Kadmon, R. (2006). Assessing the accuracy of species distribution models: Prevalence, kappa and the true skill statistic (TSS). *Journal of Applied Ecology*. https://doi.org/10.1111/j.1365-2664.2006.01214.x

Araújo, M. B., & New, M. (2007). Ensemble forecasting of species distributions. Trends in ecology & evolution, 22(1), 42-47.

Ashrafzadeh, M. R., Khosravi, R., Mohammadi, A., Naghipour, A. A., Khoshnamvand, H., Haidarian, M., & Penteriani, V. (2022). Modeling climate change impacts on the distribution of an endangered brown bear population in its critical habitat in Iran. *Science of the Total Environment*. https://doi.org/10.1016/j.scitotenv.2022.155753

Atmiş, E. (2018). A critical review of the (potentially) negative impacts of current protected area policies on the nature conservation of forests in Turkey. *Land Use Policy*. https://doi.org/10.1016/j.landusepol.2017.10.054

Barbet-Massin, M., Jiguet, F., Albert, C. H., & Thuiller, W. (2012). Selecting pseudo-absences for species distribution models: How, where and how many? *Methods in Ecology and Evolution*. https://doi.org/10.1111/j.2041-210X.2011.00172.x

Bautista, C., Oeser, J., Kuemmerle, T., & Selva, N. (2023). Resource pulses and human–wildlife conflicts: linking satellite indicators and ground data on forest productivity to predict brown bear damages. *Remote Sensing in Ecology and Conservation*. https://doi.org/10.1002/rse2.302

Breiman, L., 2001. Random forests. Machine learning, 45, pp.5-32.

Canturk, U., & Kulaç, Ş. (2021). The effects of climate change scenarios on Tilia ssp. in Turkey. *Environmental Monitoring and Assessment*. https://doi.org/10.1007/s10661-021-09546-5

Caprio, J. M., & Quamme, H. A. (2006). Influence of weather on apricot, peach and sweet cherry production in the Okanagan Valley of British Columbia. *Canadian Journal of Plant Science*. https://doi.org/10.4141/P05-032

Chen, T., 2015. Xgboost: extreme gradient boosting. R package version 0.4-2, 1(4).

Çolak, A. H., & Rotherham, I. D. (2006). A review of the forest vegetation of Turkey: Its status past and present and its future conservation. *Biology and Environment*. https://doi.org/10.3318/BIOE.2006.106.3.343

Dagtekin, D., Ertürk, A., Sommer, S., Ozgul, A., & Soyumert, A. (2024). Seasonal habitat-use patterns of large mammals in a human-dominated landscape. *Journal of Mammalogy*. https://doi.org/10.1093/jmammal/gyad107

Dai, Y., Peng, G., Wen, C., Zahoor, B., Ma, X., Hacker, C. E., & Xue, Y. (2021). Climate and land use changes shift the distribution and dispersal of two umbrella species in the Hindu Kush Himalayan region. *Science of the Total Environment*. https://doi.org/10.1016/j.scitotenv.2021.146207

Dar, S. A., Singh, S. K., Wan, H. Y., Kumar, V., Cushman, S. A., & Sathyakumar, S. (2021). Projected climate change threatens Himalayan brown bear habitat more than human land use. *Animal Conservation*. https://doi.org/10.1111/acv.12671

De'Ath, G., 2007. Boosted trees for ecological modeling and prediction. Ecology, 88(1), pp.243-251.

Deacy, W. W., Armstrong, J. B., Leacock, W. B., Robbins, C. T., Gustine, D. D., Ward, E. J., Erlenbach, J. A., & Stanford, J. A. (2017). Phenological synchronization disrupts trophic interactions between Kodiak brown bears and salmon. *Proceedings of the National Academy of Sciences of the United States of America*. <https://doi.org/10.1073/pnas.1705248114>

Demircan, M., Gürkan, H., Eskioğlu, O., Arabacı, H., & Coşkun, M. (2017). Climate Change Projections for Turkey: Three Models and Two Scenarios. *Turkish Journal of Water Science and Management*. https://doi.org/10.31807/tjwsm.297183

Ergüner, Y., Kumar, J., Hoffman, F. M., Dalfes, H. N., & Hargrove, W. W. (2019). Mapping ecoregions under climate change: a case study from the biological ‘crossroads’ of three continents, Turkey. *Landscape Ecology*. https://doi.org/10.1007/s10980-018-0743-8

Feng, X., Park, D. S., Walker, C., Peterson, A. T., Merow, C., & Papeş, M. (2019). A checklist for maximizing reproducibility of ecological niche models. *Nature Ecology and Evolution*. https://doi.org/10.1038/s41559-019-0972-5

Fick, S. E., & Hijmans, R. J. (2017). WorldClim 2: new 1-km spatial resolution climate surfaces for global land areas. *International Journal of Climatology*. https://doi.org/10.1002/joc.5086

García-Mozo, H., Dominguez-Vilches, E., & Galán, C. (2012). A model to account for variations in holm-oak (Quercus ilex subsp. ballota) acorn production in southern Spain. *Annals of Agricultural and Environmental Medicine*.

GOLDSTEIN, M. I., POE, A. J., SURING, L. H., NIELSON, R. M., & MCDONALD, T. L. (2010). Brown Bear Den Habitat and Winter Recreation in South‐Central Alaska. *The Journal of Wildlife Management*. https://doi.org/10.2193/2008-490

González-Bernardo, E., Russo, L. F., Valderrábano, E., Fernández, Á., & Penteriani, V. (2020). Denning in brown bears. In *Ecology and Evolution*. https://doi.org/10.1002/ece3.6372

Gür, H. (2022). The future impact of climate and land-use changes on Anatolian ground squirrels under different scenarios. *Ecological Informatics*. https://doi.org/10.1016/j.ecoinf.2022.101693

Hao, T., Elith, J., Guillera-Arroita, G., & Lahoz-Monfort, J. J. (2019). A review of evidence about use and performance of species distribution modelling ensembles like BIOMOD. In *Diversity and Distributions*. https://doi.org/10.1111/ddi.12892

Kemahlı, MÇA, Kusak J, Chynoweth M, Çoban E, Çoban A, Naderi M, et al. Genetic changes influence behavioral shifts of brown bears in response to human-dominated landscapes in Eastern Türkiye. 2023. Available from: <https://www.researchsquare.com/article/rs-3332392/v1>

Kennedy, C. M., J. R. Oakleaf, D. M. Theobald, S. Baruch-Mordo, and J. Kiesecker. 2020. Global Human Modification of Terrestrial Systems. Palisades, New York: NASA Socioeconomic Data and Applications Center (SEDAC). [https://doi.org/10.7927/edbc-3z60. Accessed 01.07.2024](https://doi.org/10.7927/edbc-3z60.%20Accessed%2001.07.2024).

Kozakai, C., Yamazaki, K., Nemoto, Y., Nakajima, A., Koike, S., Abe, S., Masaki, T., & Kaji, K. (2011). Effect of mast production on home range use of Japanese black bears. *Journal of Wildlife Management*. https://doi.org/10.1002/jwmg.122

Lamb, C. T., Mowat, G., McLellan, B. N., Nielsen, S. E., & Boutin, S. (2017). Forbidden fruit: human settlement and abundant fruit create an ecological trap for an apex omnivore. *Journal of Animal Ecology*. https://doi.org/10.1111/1365-2656.12589

Lehner, B., & Grill, G. (2013). Global river hydrography and network routing: Baseline data and new approaches to study the world’s large river systems. *Hydrological Processes*. https://doi.org/10.1002/hyp.9740

Lek, S. and Guégan, J.F., 1999. Artificial neural networks as a tool in ecological modelling, an introduction. Ecological modelling, 120(2-3), pp.65-73.

McCullagh, P., 2019. Generalized linear models. Routledge.

Mohammadi, A., Almasieh, K., Nayeri, D., Ataei, F., Khani, A., López-Bao, J. V., Penteriani, V., & Cushman, S. A. (2021). Identifying priority core habitats and corridors for effective conservation of brown bears in Iran. *Scientific Reports*. https://doi.org/10.1038/s41598-020-79970-z

Mukherjee, T., Sharma, L. K., Kumar, V., Sharief, A., Dutta, R., Kumar, M., Joshi, B. D., Thakur, M., Venkatraman, C., & Chandra, K. (2021). Adaptive spatial planning of protected area network for conserving the Himalayan brown bear. *Science of the Total Environment*. https://doi.org/10.1016/j.scitotenv.2020.142416

Naimi, B., Capinha, C., Ribeiro, J., Rahbek, C., Strubbe, D., Reino, L., & Araújo, M. B. (2022). Potential for invasion of traded birds under climate and land-cover change. *Global Change Biology*. https://doi.org/10.1111/gcb.16310

Naimi, B., 2023. Package “usdm” Title Uncertainty Analysis for Species Distribution Models. CRAN.

NCNP, 2022. Ministry of Agriculture and Forestry, General Directorate of and Nature Conservation and National Parks. Protected Area Statistics. Accessed 01.10.2024. https://www.tarimorman.gov.tr/DKMP/Belgeler/Tabiat%20Koruma%20Durum%20Raporu/TKDR_EN_2022.pdf

Noroozi, J., Zare, G., Sherafati, M., Mahmoodi, M., Moser, D., Asgarpour, Z., & Schneeweiss, G. M. (2019). Patterns of endemism in Turkey, the meeting point of three global biodiversity hotspots, based on three diverse families of vascular plants. *Frontiers in Ecology and Evolution*. https://doi.org/10.3389/fevo.2019.00159

Ozturk, T., Ceber, Z. P., Türkeş, M., & Kurnaz, M. L. (2015). Projections of climate change in the Mediterranean Basin by using downscaled global climate model outputs. *International Journal of Climatology*. https://doi.org/10.1002/joc.4285

Pant, G., Maraseni, T., Apan, A., & Allen, B. L. (2021). Predicted declines in suitable habitat for greater one-horned rhinoceros (Rhinoceros unicornis) under future climate and land use change scenarios. *Ecology and Evolution*. https://doi.org/10.1002/ece3.8421

Parsons, B., Wilson, A. E., Graham, K., & Stenhouse, G. B. (2023). Grizzly bear (Ursus arctos) movements and habitat use predict human-caused mortality across temporal scales. *Canadian Journal of Zoology*. https://doi.org/10.1139/cjz-2022-0054

Penteriani, V., Zarzo-Arias, A., Novo-Fernández, A., Bombieri, G., & López-Sánchez, C. A. (2019). Responses of an endangered brown bear population to climate change based on predictable food resource and shelter alterations. *Global Change Biology*. https://doi.org/10.1111/gcb.14564

Pérez-Girón, J. C., Álvarez-Álvarez, P., Ballesteros, F., & López-Bao, J. V. (2024). Potential impacts of climate change on wild cherry distribution and associated consequences on brown bears. *Biological Conservation*. https://doi.org/10.1016/j.biocon.2023.110390

Pérez-Girón, J. C., Díaz-Varela, E. R., Álvarez-Álvarez, P., Hernández Palacios, O., Ballesteros, F., & López-Bao, J. V. (2022). Linking landscape structure and vegetation productivity with nut consumption by the Cantabrian brown bear during hyperphagia. *Science of the Total Environment*. https://doi.org/10.1016/j.scitotenv.2021.152610

Phillips, S. J., Anderson, R. P., Dudík, M., Schapire, R. E., & Blair, M. E. (2017). Opening the black box: an open-source release of Maxent. *Ecography*. <https://doi.org/10.1111/ecog.03049>

Piédallu, B., Quenette, P. Y., Bombillon, N., Gastineau, A., Miquel, C., & Gimenez, O. (2019). Determinants and patterns of habitat use by the brown bear Ursus arctos in the French Pyrenees revealed by occupancy modelling. *ORYX*. https://doi.org/10.1017/S0030605317000321

Pigeon, K. E., Stenhouse, G., & Côté, S. D. (2016). Drivers of hibernation: linking food and weather to denning behaviour of grizzly bears. *Behavioral Ecology and Sociobiology*. https://doi.org/10.1007/s00265-016-2180-5

Recio, M. R., Knauer, F., Molinari-Jobin, A., Huber, Filacorda, S., & Jerina, K. (2021). Context-dependent behaviour and connectivity of recolonizing brown bear populations identify transboundary conservation challenges in Central Europe. *Animal Conservation*. https://doi.org/10.1111/acv.12624

Roberts, D. R., Nielsen, S. E., & Stenhouse, G. B. (2014). Idiosyncratic responses of grizzly bear habitat to climate change based on projected food resource changes. *Ecological Applications*. https://doi.org/10.1890/13-0829.1

Roever, C. L., Boyce, M. S., & Stenhouse, G. B. (2010). Grizzly bear movements relative to roads: Application of step selection functions. *Ecography*. https://doi.org/10.1111/j.1600-0587.2010.06077.x

Schiavina, M., Melchiorri, M., Pesaresi, M. (2023): GHS-POP R2023A - GHS population grid, multitemporal (1975-2030). European Commission, Joint Research Centre (JRC) [Dataset]. DOI: 10.2905/A0DF7A6F-49DE-46EA-9BDE-563437A6E2BA

Şekercioĝlu, Ç. H., Anderson, S., Akçay, E., Bilgin, R., Can, Ö. E., Semiz, G., Tavşanoĝlu, Ç., Yokeş, M. B., Soyumert, A., Ipekdal, K., Saĝlam, I. K., Yücel, M., & Nüzhet Dalfes, H. (2011). Turkey’s globally important biodiversity in crisis. In *Biological Conservation*. <https://doi.org/10.1016/j.biocon.2011.06.025>

Sharma, P., Gurung, J., Wangchuk, K., Uddin, K., & Chettri, N. (2022). Changing Landscape and Escalating Human-Wildlife Conflict: Introspection from a Transboundary Landscape. In Conservation, Management and Monitoring of Forest Resources in India (pp. 459-476). Cham: Springer International Publishing.

Sıkdokur, E., Naderi, M., Çeltik, E., Aytekin, M. Ç. K., Kusak, J., Sağlam, İ. K., & Şekercioğlu, Ç. H. (2024). Human-brown bear conflicts in Türkiye are driven by increased human presence around protected areas. Ecological Informatics, 81, 102643.

Sillero, N., Arenas-Castro, S., Enriquez‐Urzelai, U., Vale, C. G., Sousa-Guedes, D., Martínez-Freiría, F., Real, R., & Barbosa, A. M. (2021). Want to model a species niche? A step-by-step guideline on correlative ecological niche modelling. *Ecological Modelling*. https://doi.org/10.1016/j.ecolmodel.2021.109671

Sillero, N., & Barbosa, A. M. (2021). Common mistakes in ecological niche models. In *International Journal of Geographical Information Science*. https://doi.org/10.1080/13658816.2020.1798968

Su, J., Aryal, A., Hegab, I. M., Shrestha, U. B., Coogan, S. C. P., Sathyakumar, S., Dalannast, M., Dou, Z., Suo, Y., Dabu, X., Fu, H., Wu, L., & Ji, W. (2018). Decreasing brown bear (Ursus arctos) habitat due to climate change in Central Asia and the Asian Highlands. *Ecology and Evolution*. https://doi.org/10.1002/ece3.4645

Suel, H. (2019). Brown bear (Ursus Arctos) habitat suitability modelling and mapping. *Applied Ecology and Environmental Research*. https://doi.org/10.15666/aeer/1702_42454255

Sulla-Menashe, D., & Friedl, M. A. (2018). MCD12Q1 MODIS/Terra+Aqua Land Cover Type Yearly L3 Global 500m SIN Grid V006 [Data set]. NASA EOSDIS Land Processes DAAC. doi:10.5067/MODIS/MCD12Q1.006.

Taşoğlu, E., Öztürk, M. Z., & Yazıcı, Ö. (2024). High Resolution Köppen‐Geiger Climate Zones of Türkiye. International Journal of Climatology, 44(14), 5248-5265.

Thuiller, W., Guéguen, M., Renaud, J., Karger, D. N., & Zimmermann, N. E. (2019). Uncertainty in ensembles of global biodiversity scenarios. *Nature Communications*. https://doi.org/10.1038/s41467-019-09519-w

Thuiller, W., Lafourcade, B., Engler, R., & Araújo, M. B. (2009). BIOMOD - A platform for ensemble forecasting of species distributions. *Ecography*. https://doi.org/10.1111/j.1600-0587.2008.05742.x

Wei, T., Simko, V.R., Levy, M., Xie, Y., Jin, Y. and Zemla, J., 2021. package “corrplot”: Visualization of a Correlation Matrix. 2017. Version 0.84.

Zarzo-Arias, A., Delgado, M. M., Palazón, S., Afonso Jordana, I., Bombieri, G., González-Bernardo, E., Ordiz, A., Bettega, C., García-González, R., & Penteriani, V. (2021). Seasonality, local resources and environmental factors influence patterns of brown bear damages: implications for management. *Journal of Zoology*. https://doi.org/10.1111/jzo.12839

Zarzo-Arias, Alejandra, Penteriani, V., Del Mar Delgado, M., Torre, P. P., García-González, R., Mateo-Sánchez, M. C., García, P. V., & Dalerum, F. (2019). Identifying potential areas of expansion for the endangered brown bear (Ursus arctos) population in the cantabrian mountains (NW Spain). *PLoS ONE*. https://doi.org/10.1371/journal.pone.0209972

Ziółkowska, E., Ostapowicz, K., Radeloff, V. C., Kuemmerle, T., Sergiel, A., Zwijacz-Kozica, T., Zięba, F., Śmietana, W., & Selva, N. (2016). Assessing differences in connectivity based on habitat versus movement models for brown bears in the Carpathians. *Landscape Ecology*. https://doi.org/10.1007/s10980-016-0368-8.
